# Cardiac implantable electronic device upgrades and downgrades: a *Clinical Consensus Statement* of the European Heart Rhythm Association (EHRA) of the ESC, the Asia Pacific Heart Rhythm Association (APHRS), Canadian Heart Rhythm Society (CHRS), Heart Rhythm Society (HRS), and the Latin American Heart Rhythm Society (LAHRS)

**DOI:** 10.1093/europace/euaf252

**Published:** 2025-12-11

**Authors:** Daniel Keene, Jens Cosedis Nielsen, Haran Burri, Carlos Alejandro Chavez-Gutierrez, Jean-Claude Deharo, Inga Drossart, James E Ip, Carsten W Israel, Jens Brock Johansen, Annamaria Kosztin, Chu-Pak Lau, Shuli Levy, Jaimie Manlucu, Lina Marcantoni, Margarida Pujol-Lopez, Archana Rao, Christoph Starck, Jose Maria Tolosana, Lieselot Van Erven, Julia Vogler, Nandita Kaza

**Affiliations:** National Heart and Lung Institute, Imperial College London, London, UK; Department of Cardiology, Imperial College Healthcare NHS Trust, London, UK; Department of Cardiology, Royal Free London NHS Foundation Trust, London, UK; Department of Cardiology, Aarhus University Hospital, Aarhus, Denmark; Department of Clinical Medicine, Aharhus University, Aarhus, Denmark; Cardiac Pacing Unit, Cardiology Department, University Hospital of Geneva, Rue Gabrielle Perret Gentil 4, Geneva 14 1211, Switzerland; Department of Cardiology, National Medical Centre of the West, Guadalajara, Jalisco, Mexico; Department of Cardiology, Real San Jose Valle Real Hospital, Zapopan, Jalisco, Mexico; Department of Cardiology, L'hôpital de la Timone, Marseille 13005, France; European Society of Cardiology, Sophia Antipolis, France; ESC Patient Forum, Sophia Antipolis, France; Weill Cornell Medicine, New York Presbyterian Hospital, New York, NY, USA; Department of Medicine—Cardiology, Bethel-Clinic, Bielefeld, Germany; Department of Cardiology, Odense University Hospital, Odense, Denmark; Heart and Vascular Center, Semmelweis University, Budapest, Hungary; Queen Mary Hospital, The University of Hong Kong, Hong Kong SAR, China; Department of Geriatrics and General Internal Medicine, Imperial College Healthcare NHS Trust, London, UK; Department of Cardiology, Western University/London Health Sciences Centre, London, Ontario, Canada; Department of Cardiology, Santa Maria Della Misericordia Hospital, Rovigo, Italy; Institut Clínic Cardiovascular (ICCV), Hospital Clínic, Universitat de Barcelona, Institut D’Investigacions Biomèdiques August Pi I Sunyer (IDIBAPS), Barcelona, Catalonia, Spain; Department of Cardiology, Liverpool Heart and Chest Hospital, Liverpool, UK; Department of Cardiothoracic and Vascular Surgery, German Heart Center of Charité, Berlin, Germany; Charité—Universitaetsmedizin Berlin, corporate Member of Freie Universitaet Berlin and Humboldt Universitaet zu Berlin, Berlin, Germany; DZHK (German Centre for Cardiovascular Research), partner site Berlin, Germany; Institut Clínic Cardiovascular (ICCV), Hospital Clínic, Universitat de Barcelona, Institut D’Investigacions Biomèdiques August Pi I Sunyer (IDIBAPS), Barcelona, Catalonia, Spain; Department of Cardiology, Centro de Investigación Biomédica en Red—Enfermedades Cardiovasculares (CIBERCV), Madrid, Spain; Department of Cardiology, Leiden University Medical Center, Leiden, The Netherlands; Department of Cardiology and Intensive Care Medicine, Asklepios Klinik St. Georg, Hamburg, Germany; Department of Arrhythmology, University Hospital Schleswig-Holstein Campus Lübeck, Lubeck, Germany; National Heart and Lung Institute, Imperial College London, London, UK; Department of Cardiology, Imperial College Healthcare NHS Trust, London, UK

**Keywords:** CIED upgrade, Downgrade

## Abstract

Cardiac implantable electronic device upgrade and downgrade procedures are increasingly being performed. Whilst the most appropriate guideline-recommended device may have been followed during a patient's initial procedure, the requirements of patients can change over time. This could be due to worsening of cardiac function due to detrimental effects of pacing itself or the diagnosis, development, or progression of another cardiac comorbidity. Device downgrades are also performed when a patient's clinical state changes and are often considered in patients with increased frailty and comorbidity. This clinical consensus statement aims to provide a framework for screening patients for device upgrade, pre-procedural planning considerations, available procedural strategies, namely a summary of techniques and approaches for vascular access, including ipsilateral and contralateral options, and a framework for when extraction to gain access may be appropriate. The document also provides advice on how to frame an ethical discussion with patients and carers on available options.

## Graphical Abstract

**Graphical Abstract:**
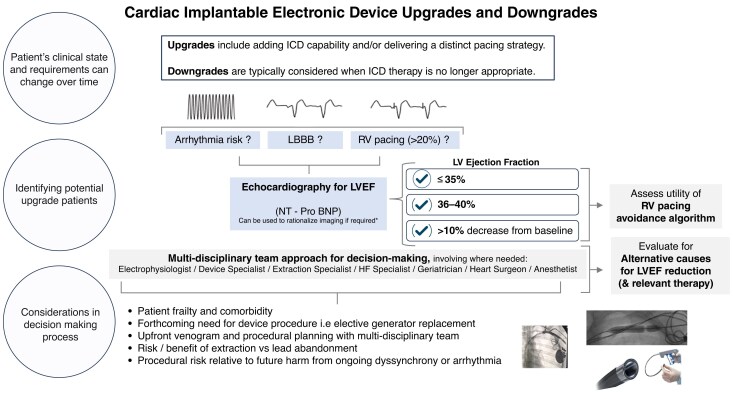

## Table of contents

Introduction
 Overview of clinical data and guidelines
  Summary of potential procedural risk and benefit
Effective workflow and approach to practice
 Optimizing initial choice of cardiac implantable electronic device
 Screening for eligible patients
 Timing of device upgrades or downgrades
 Shared decision-making
 Hardware considerations during decision-making
 Operator experience and need for specialist skills
 Pre-procedural steps–importance of procedural planning and contingency planning
 Value of pre-procedural cardiac imaging
  Determining the need for upgrade–assessment of left ventricular function
  Assessment of tricuspid regurgitation
 Additional utilization of imaging
  Cardiac computerized tomography
  Cardiac magnetic resonance imaging
  Upfront superior vascular anatomy assessment: the venogram
 Anaesthetic considerations
 Medication considerations
  Anticoagulation and antiplatelets
 Equipment and lab set-up considerations
Vascular superior access considerations
 Vascular access issues
 Techniques to manage venous stenoses/obstruction
 Vascular superior access considerations
  Preparation
  Puncture
 Access through an occluded vessel
 Serial dilatation
 Balloon venoplasty
 Infrequently used methods
 Contralateral new lead implantation and tunnelling/contralateral new system implant and abandonment of original system
 Lead extraction to create venous access or manage redundant leads
Alternative (non-superior access) delivery approaches
 Surgical/epicardial approach
 Leadless, endocardial, and other non-transvenous approaches
 Femoral approach for lead placement
Surgical considerations
 Consideration 1: timing of skin incision and pocket opening in relation to vascular access for cardiac implantable electronic device lead placement
 Consideration 2: location of incision
 Consideration 3: surgical techniques; blunt dissection vs. diathermy vs. low-thermal-injury dissection device
 Consideration 4: capsulectomy
 Consideration 5: management of abandoned leads
 Consideration 6: infection prevention and antibiotic envelopes (Table 7)
 Antibiotic envelopes
 Patient perspective
Considerations for individualized risk–benefit analysis
 Suggested framework
  Role of the multi-disciplinary team and ethical framework
  Risk at generator change vs. any other time
  Specific situations
   Device deactivation
Distinct case scenario discussions
Device downgrade considerations
 Downgrade of cardiac resynchronization therapy defibrillator to cardiac resynchronization therapy pacemaker
  Practical considerations
 Downgrade of implantable cardioverter defibrillator to pacemaker
  Practical considerations
 Downgrade from dual-chamber to single-chamber device
 When a cardiac implantable electronic device is no longer indicated
Distinct patient scenarios
Conclusion

## Introduction

Cardiac implantable electronic device (CIED) upgrade and downgrade procedures are increasingly being performed in clinical practice. Whilst the most appropriate guideline-recommended device may have been used during a patient's initial implant procedure, the requirement of patients can change over time. In these scenarios, the risks and benefits of changing the capabilities of a device need to be carefully considered prior to further device interventions.

Device upgrades frequently include the addition of cardiac resynchronization therapy (CRT) capabilities [biventricular pacing (BiV) or, more recently, conduction system pacing (CSP)], defibrillator capabilities, or both, or delivery of dual-chamber pacing capabilities with the addition of an atrial lead. The desire to upgrade a device can result from cardiac dysfunction due to detrimental effects of pacing itself or the development, discovery, or progression of another cardiac comorbidity.

Furthermore, patients are living longer, and it is becoming increasingly frequent to encounter patients in whom the initially implanted device is no longer appropriate, e.g. patients with a previous defibrillator indication who have developed advanced frailty in whom defibrillator replacement may no longer be an appropriate or desired strategy. In such patients, clinical decision-making can be challenging, particularly in those with a continued need for ventricular pacing (namely in those with an implanted DF-4 lead).^1–4^

The objective of this clinical consensus statement is to provide advice on how to optimally assess, plan, and perform device upgrades and downgrades with consideration for both pre-procedural aspects as well as procedural techniques. This includes discussion of effective and safe clinical practice and an appraisal of the toolkit of techniques that can be utilized.

The document is based upon both published evidence and expert consensus with the intention to standardize procedural approaches, improve success rates, avoid complications, and ultimately improve patient outcomes.

In this clinical consensus statement, different categories of advice and the respective definitions are presented in *Table 1*. Furthermore, the evidence supporting each advice has been classified in different categories based on the type, quality, and quantity of respective sources (*Tables 2* and *3*).

**Table 1:** Definition of categories of advice and areas of uncertainty

Definition	Categories of advice
Evidence or general agreement that a given measure is clinically useful and appropriate	Advice TO DO
Evidence or general agreement that a given measure may be clinically useful and appropriate	May be appropriate TO DO
Evidence or general agreement that a given measure is not appropriate or harmful	Advice NOT TO DO
No advice can be given because of lack of data or inconsistency of data. The topic is important to be addressed	Areas of uncertainty

**Table 2:** Type and strength of supporting evidence

Type of supporting evidence	Strength of evidence
Published data^a^	>1 high-quality RCT Meta-analysis of high-quality RCT
	High-quality RCT > 1 moderate quality RCT Meta-analysis of moderate quality RCT
	High quality, large observational studies
Expert opinion^b,c^	Strong consensus >90% of writing group (WG) supports advice
	Consensus >70% of WG supports advice

RCT, randomized controlled trial; WG, writing group.

^a^The reference for the published data that fulfil the criteria is indicated in the table of advice, if applicable.

^b^Expert opinion also considers: Randomized, non-randomized, observational, or registry studies with limitations of design or execution, case series, meta-analyses of such studies, and physiological or mechanistic studies in human subjects.

^c^For areas of uncertainty strong consensus/consensus that the topic is relevant and important to be addressed by future trials.

**Table 3:** Example of a table of advice

Definition and delineation of the target	Strength of evidence
**Advised TO DO**	
It is advised to, it is appropriate, it is useful	>1 high-quality RCT Meta-analysis of high-quality RCT
It is advised to, it is appropriate, it is useful	High-quality RCT > 1 moderate quality RCT Meta-analysis of moderate quality RCT
It is advised to, it is appropriate, it is useful	>90% of writing group agree
It is advised to, it is appropriate, it is useful	>70% of writing group agree
**May be appropriate TO DO**	
It may be appropriate, it may be useful	High quality, large observational studies
It may be appropriate, it may be useful	>90% of writing group agree
**Advised NOT TO DO**	
It is not advised, it is not appropriate, it is harmful	>90% of writing group agree
**Areas of uncertainty**	
It is unknown, lack of data, inconsistency of data	>90% of writing group agree
It is uncertain, lack of data, inconsistency of data	>70% of writing group agree

All clinical consensus statements were subject to voting and required a >70% consensus by the writing group in order to be adopted.

### Overview of clinical data and guidelines

Up to ∼30% of patients living with an implanted pacemaker or an implantable cardioverter defibrillator (ICD) may develop significant deterioration in left ventricular (LV) systolic function with associated adverse clinical outcomes and heart failure (HF) induced or worsened by right ventricular (RV) pacing.^5–9^

The guidelines for CIED upgrade in patients with RV pacing to more physiological pacing approaches differ across societies and have changed in both level of recommendation and the defined patient cohort over the last 10 years^10–15^ (*Table 4*). Since the 2021 European Society of Cardiology (ESC) guidelines were published, additional relevant randomized controlled trial (RCT) and meta-analysis data have also been published.

**Table 4:** Summary of available and prior guideline recommendations for device upgrade procedures

	Prior guideline	Current guideline
ESC (EHRA) Guidelines	2013 Recommendation for BiV upgrade if LVEF < 35% despite OMT and significant proportion of RVP	2021 Recommendation for BiV upgrade if LVEF < 35% despite OMT and significant proportion of RVP
	*The ESC 2021 pacing guidelines highlight a knowledge gap in this area*.^13^ *The ESC 2021 pacing guideline was published prior to Budapest-CRT publications and recent meta-analyses of available data* *No comment is made on upgrade to conduction system pacing devices in the guideline, however, upgrade considerations are included in the 2025 ESC/EHRA consensus on CSP indications*^4^	
HRS/LAHRS/APHRS Guidelines	2012 No formal recommendation regarding device upgrade. However, the guideline ‘recognized the ability to improve LV dysfunction in the setting of chronic RV pacing with an upgrade to a biventricular device’^10^	2023 Recommendation for device upgrade to biventricular pacing. Recommendation for upgrade to CSP.^14^ *Relevant patients are defined as those with substantial RVP and any decline in left ventricular function or symptoms noted, suggesting that device upgrade should not necessarily be restricted to only those with severely impaired ejection fraction*
Canadian HRS Guidelines	2014 Weak Recommendation CRT may be considered for patients with chronic RV pacing or who are likely to be chronically paced, have signs or symptoms of heart failure and a LVEF ≤ 35%	*(No guideline update since 2014)*

LVEF, left ventricular ejection fraction; OMT, optimal medical therapy; RVP, right ventricular pacing; BiV, biventricular; CSP, conduction system pacing

The clinical data available on device upgrades are largely based on observational datasets as well as small (predominantly crossover) trials. Much of the data focuses on cohorts with severe impairment of LV function. Data on upgrades in patients with less severe LV impairment or PICM are more limited. Meta-analyses of these data do, however, show improvements in left ventricular ejection fraction (LVEF), left ventricular end-systolic volume (LVESV), brain-natriuretic peptide (BNP), QRS duration, and quality of life.^16–25^ These studies were not designed to detect differences in hospitalization or mortality.

Although LVEF and NT-proBNP are not formally validated surrogate endpoints for hard clinical outcomes, and their prognostic value can vary across patient groups, they remain amongst the most widely reported and biologically plausible indicators of response in device studies. In the absence of multiple large, event-driven randomized trials in this space, improvements in these markers (particularly when reflective of reverse remodelling) offer valuable insight into the mechanistic and potential clinical benefit of therapy. In the CARE-HF trial of CRT, for example, CRT was associated with significant improvements in LVEF and NT-proBNP, which paralleled reductions in mortality and HF hospitalizations.^26^ Similar associations were seen in the BUDAPEST-CRT Upgrade Trial, where reverse remodelling (including a change in LVEF) occurred alongside substantial reductions in the composite of mortality and HF hospitalization.^27,28^

The BUDAPEST-CRT Upgrade Trial results were published after the most recent ESC pacing guideline. This was the first, large, prospective, multi-centre, randomized trial to compare the efficacy and safety of a CRT upgrade, compared to ICD alone, in patients with HF and reduced ejection fraction (HFrEF) patients (LVEF ≤ 35%) on guideline-directed medical therapy (GDMT) with HF symptoms [New York Heart Association NYHA II-IVa (which consists of outpatients with NYHA class IV)], a previously implanted pacemaker or ICD and intermittent or permanent RV pacing (RVP ≥ 20%).^27,28^ A total of 360 patients were randomly assigned to cardiac resynchronization therapy defibrillator (CRT-D) upgrade (*n* = 215) or an ICD (*n* = 145). During a median of 12.4 months, the primary outcome of HF hospitalization, all-cause mortality or <15% reduction of LV end-systolic volume occurred in 58/179 (32.4%) patients in the CRT-D arm and 101/128 (78.9%) in the ICD arm [adjusted odds ratio (OR) 0.11; 95% confidence interval (CI), 0.06–0.19; *P* < 0.001] with a homogenous effect across all pre-specified subgroups. In the composite of HF hospitalization and all-cause mortality, CRT-D showed a substantial benefit compared to ICD (adjusted hazard ratio 0.28, 95% CI 0.17–0.46; *P* < 0.001). Left ventricular morphological and functional response according to echocardiography also favoured CRT-D compared to ICD. While the inclusion criteria for the trial was of Vp (ventricular pacing) >20%, the reported pacing burden was much higher (Vp 85.4 ± 21.1% for CRT-D vs. Vp 88.1 ± 18.8% in controls). Whilst a greater RV pacing percentage might be associated with greater potential benefit; trials and observational studies have frequently used an arbitrary cutoff of >20% ventricular pacing burden with associated benefit.^5–7,13,29^

No RCT of upgrade to CSP has been completed, although one is in progress (PROTECT-UP, NCT06052475). Several meta-analyses of observational studies of CSP upgrade (both to His bundle and left bundle branch area pacing) suggest similar improvements in LVEF, LVESV, and NYHA class compared to studies of biventricular pacing. These results are similarly observed in cohorts without severe LV impairment.^16,30^ Furthermore, trials are ongoing comparing CSP with other pacing modes including biventricular pacing and RV pacing and these results will be essential in further defining the role of CSP.

#### Summary of potential procedural risk and benefit

Due to the complexity of CIED upgrade or downgrade procedures, individual assessment and consideration of the patients’ risk–benefit profile are crucial.

Patients referred for upgrades are frequently older with a higher burden of comorbidities compared to those receiving *de novo* devices. This, coupled with advanced age and frailty, can affect procedural outcomes.^31–35^ Procedural risks also increase with the complexity of the approach and the number of leads involved. For a given patient, individualized risk assessment is required to determine, for example, whether it is preferable to abandon a redundant lead or extract it. Patients undergoing removal of an unused or malfunctioning ICD lead might have higher rates of in-hospital complications than those with a lead abandonment strategy alone; however, an abandoned lead may, albeit at low risk, have unpredictable delayed sequalae e.g. interference with new leads, complications associated with abandoned leads and the potential for more complex and higher-risk extraction if needed in the future due to longer lead dwell time downstream.^36,37^

One of the most severe complications is device infection. The incidence is highest after CIED replacements or upgrades when compared to other device procedures and is associated with poor outcomes.^38^ The frequency of complications is also associated with operator experience, highlighting the relevance of referral of such patients to high-volume experienced centres.^39^ In registry data, complication rates in general are seen to be up to 2–3 times higher for upgrade procedures than *de novo* implantations.^40–43^ These include the risk of lead-related intervention due to displacement, infection, pocket haematoma, pneumothorax, cardiac perforation, deep vein thrombosis, tricuspid regurgitation (TR), and pain or discomfort requiring revision.

**Figure 1:**
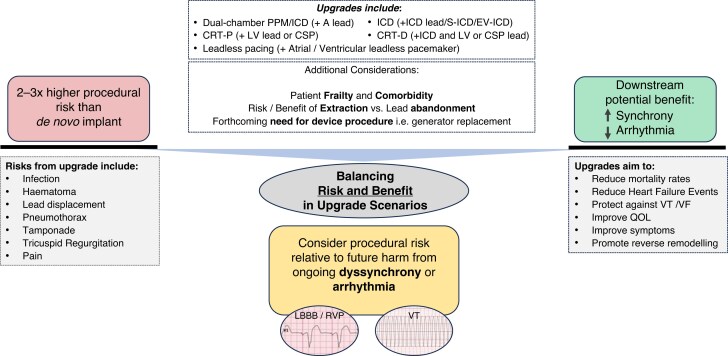
Balancing whether to perform a device upgrade needs careful consideration of the upfront procedural risk against the likely potential future benefit (PPM, pacemaker; A lead, atrial lead; CRT-P, cardiac resynchronization therapy pacemaker; LV, left ventricular; CSP, conduction system pacing; ICD, implantable cardioverter defibrillator; QOL, quality of life; VT, ventricular tachycardia; VF, ventricular fibrillation).

Procedural risk should not necessarily be compared to *de novo* procedures in these scenarios, but rather balanced against the potential benefit to be gained by the patient by upgrading from the ongoing current pacing approach. Examples where intervening can be associated with significant benefit could include scenarios such as the development of bradycardia requiring atrial pacing in a patient with a VVI ICD or the development of left bundle branch block (LBBB) and a need for CRT pacing in a patient with an initial dual-chamber ICD implant. *Figure 1* highlights the considerations to be taken into account when determining whether to upgrade or not for a given patient.

## Effective workflow and approach to practice

### Optimizing initial choice of cardiac implantable electronic device

Following guideline-directed investigations and comprehensive patient evaluation can help ensure the most appropriate CIED is selected at the time of initial implantation. For example, in a patient under 60 years old presenting with new-onset 2:1 atrioventricular (AV) block (with anticipated high burden of pacing), cardiac MRI showing LVEF < 50% and a mid-septal scar pattern may suggest an underlying cardiomyopathy such as Lamin A/C disease. In such a scenario, upfront implantation of a CRT-D may be preferable to a dual-chamber pacemaker, potentially avoiding the need for future upgrade procedures.

Nonetheless, initial device selection often occurs in the context of evolving or incomplete clinical information. In many cases, the absence of clear disease-specific markers or definitive imaging findings limits the ability to fully risk-stratify patients at the time of initial implant. As a result, even when best-practice recommendations are followed, the progression of underlying disease may necessitate device upgrade at a later stage.

### Screening for eligible patients

At all planned device follow-up visits, assessment as to whether a patient has the most appropriate device implanted should be performed. Consideration should be given to whether any changes in device programming can be used to improve cardiac function. This may involve consideration of utilizing pacing avoidance algorithms if not already in use.^44^ It is worth noting, however, though that for some patients, pacing avoidance algorithms, which allow long AV delays, may be of equal harm to high burden RV pacing, and may be associated with acute deterioration of cardiac function and higher incidence of atrial fibrillation.^45,46^

With regard to device upgrades, assessment may include a review of patient-reported symptoms, hospitalizations related to cardiac issues, and pacemaker-derived data, including the burden of RV pacing, presence of arrhythmias, markers of fluid accumulation (thoracic impedance), decline in daily patient activity, or functional status. Electrocardiogram analysis may also be useful and may reveal, for example, PR interval prolongation (which could be a sign of AV dysynchrony) or QRS duration widening (a sign of interventricular dysynchrony), which may be targeted by an upgraded device for therapeutic benefit.^47^ Furthermore, the presence of sinus rhythm in a patient with a single-chamber ventricular pacemaker with signs or symptoms of pacemaker syndrome may benefit from the addition of an atrial lead to deliver AV synchrony.

Any concerns raised may then be further evaluated with appropriate follow-up testing, including measures of NT-Pro-BNP and/or an echocardiogram.

**Figure 2:**
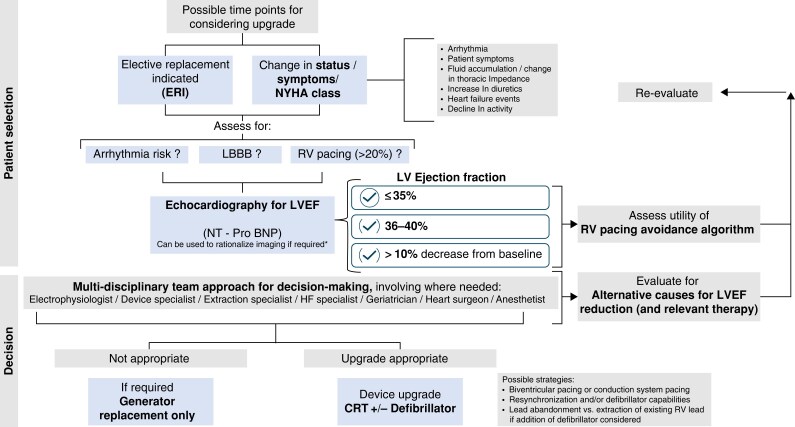
Flow chart outlining a screening approach for how to identify patients who may benefit from consideration of device upgrade utilizing symptoms and echocardiographic parameters. Caution and best clinical judgement must be exercised when evaluating echocardiographic parameters, acknowledging that there is an accepted margin of measurement error. A multi-disciplinary team approach (including specialists across professional groups with distinct areas of expertise) is suggested including where needed for example a geriatrician to assess frailty. *RCT data support the ‘advice to do’ for LVEF ≤ 35%^27^ whilst the ‘may be appropriate to do’ advice is supported by crossover studies, observational meta-analyses and other published consensus.^16,20,25,29,30,48,49^ **BNP can also be elevated for other reasons (i.e. AF or TR) and must be interpreted in clinical context.^50^

Signs suggestive of atrial fibrillation or other rhythm disturbances that may be contributing to a patient's symptoms or functional decline should be taken into account and addressed by targeted medical or ablative strategies where appropriate. This may, in some patients, avoid the need for device revision or upgrade. When patients attend for follow-up within an approximate 12-month window prior to reaching the elective replacement indicator, it may be useful for centres to adopt a more structured approach to clinical assessment. Effective pathways (as outlined in *Figure 2*) should identify suitable patients (i.e. those with a RV pacing burden >20%, or those with an ICD who later develop LBBB) who would benefit from a more detailed symptom assessment and referral for additional investigations.

It is important to note here that a potential limitation of remote CIED monitoring (now widely adopted in many centres as standard of care, in particular with patients with bradycardia devices) may be that there are reduced opportunities to conduct a comprehensive clinical patient assessment.

Echocardiography is felt to be the most useful test for evaluation of cardiac function. If resources are limited, NT-Pro-BNP can be used as a gatekeeper for imaging. These test results can then inform onward decisions regarding device upgrade.

If LVEF deterioration is confirmed, secondary causes should be investigated. This process may involve clinicians from other sub-specialties; for example, HF specialists to support investigations and optimization of GDMT or interventional cardiologists to assess alternative contributors i.e. ischaemic heart disease. In some situations, it may be pertinent to revisit the patient's initial diagnosis, for example investigating the possibility of a diagnosis of neuromuscular disease, Lamin cardiomyopathy, sodium channel disease or Anderson–Fabry disease (e.g. hypertrophic phenotype). Assessment using MRI with late-gadolinium enhancement can help to evaluate deterioration in LVEF and rework the clinical diagnosis where needed.

In those patients who present with ventricular arrhythmias or AV block at a younger age, genetic testing (which for example, may not have been available or considered at time of initial implant, or the scope of the gene panel limited in comparison to current-day options) may reveal underlying diagnoses that could offer insight to upgrade a pacemaker rather than proceeding with a generator change only (i.e. potential risk of LV dysfunction or susceptibility to ventricular arrhythmias).^51–53^ Prior to elective generator replacement, the option of device downgrades should be assessed in patients with increasing comorbidities, frailty, or non-cardiac life-limiting diagnoses, such as a new diagnosis of a terminal cancer with short life expectancy, advanced organ failure, or dementia, or a change in their goals of care for any reason. The decision to undertake a downgrade is no doubt challenging, and there is uncertainty in the optimal assessment of arrhythmic risk at different stages of underlying disease.^54^ For example, in a registry of 325 CRT-D recipients, 7% of patients had defibrillator therapy for ventricular arrhythmia after a first generator replacement despite no prior therapies.^55^ Patient's attitudes towards generator replacement at an advanced age and even in advanced illness can vary significantly, with one survey of 3067 participants stating that 55% would elect to proceed with continuation of therapy even if seriously unwell.^56^ Other studies of patient perceptions have also suggested that many patients do not realize that ICD generator non-replacement is a management option. This underscores the need for structured and careful discussions on this topic with patients when the relevant circumstances arise, explaining both the advantages and disadvantages of each strategy.

### Timing of device upgrades or downgrades

Device upgrades or downgrades can be performed at any point during a patient's device lifetime, contingent upon the clinical situation. The decision to defer a device upgrade or downgrade to the time of a generator replacement or a later undetermined date is common (even when development or progression of HF signs and symptoms are present).^57,58^ Whether this is an appropriate management approach remains unclear. The BUDAPEST-CRT RCT did, however, show a 35% rate of HF hospitalizations or death within 12 months without upgrade for patients with HF.^27,28^ This consensus statement advocates that in the presence of a change in clinical status and/or deterioration in cardiac function meeting an indication for revising an existing CIED, a device intervention should not be deferred until an elective generator replacement procedure.

Factors affecting this decision process include: additional device and procedural costs, any uncertainty of the potential benefit of an upgrade for a given patient, and an increased risk of procedural complications in upgrade procedures (6.2–20.9%).^40^ Careful shared decision-making is therefore required in order to evaluate whether the potential for benefit outweighs the procedural risk.^4,21^

### Shared decision-making

**Figure 3:**
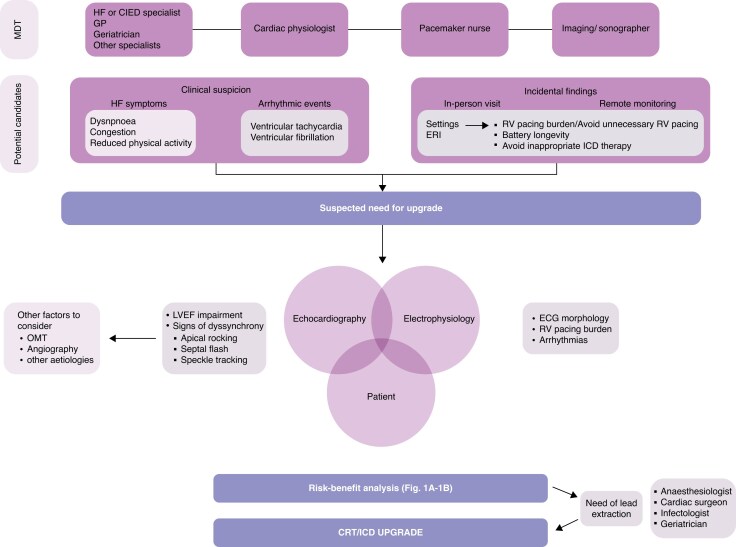
Outlines a multi-disciplinary approach for determining whether there is a clinical need for an upgrade and key findings to help guide the decision-making process (MDT, multi-disciplinary team; HF, heart failure; CIED, cardiac implantable electronic device; GP, general practitioner; ERI, elective replacement indicator; ICD, implantable cardioverter defibrillator; OMT, optimal medical therapy; LVEF, left ventricular ejection fraction; ECG, electrocardiogram; RV, right ventricular; CRT, cardiac resynchronization therapy).

A shared decision-making process should follow an integrated, patient-centred approach (*Figure 3*), which focuses on individual goals of care, patient preferences, and the specific risk–benefit considerations of each available clinical management strategy. This process will most frequently be led by a cardiologist and the patient with input from the wider multi-disciplinary team.

It should be emphasized that many device upgrades occur due to a predictable evolution of the underlying disease, which cannot be accurately captured at baseline implantation. Patients who receive CIEDs should be empowered to understand that some cardiac substrates evolve over time and it may become necessary to modify the technology used in their CIED beyond their initial implant procedure. This may help to mitigate any frustration or mistrust. An open discussion about therapeutic priorities and patients’ wishes about end-of-life management should also be undertaken during follow-up to make any eventual decisions about upgrade or downgrade less unexpected and distressing for the patient.^59^

If patients are functionally limited primarily because of worsening HF, then intervention may be encouraged sooner rather than later.^60^ However, in those who are very elderly and/or very frail with limited functional capacity and/or a short life expectancy (particularly when caused by non-cardiac issues) the benefit of an upgrade may have limited clinical impact and the incremental risk of complications related to the upgrade procedure should be carefully evaluated.

The current assessment approach relies predominately on LVEF assessment, risk stratification for susceptibility to ventricular arrhythmias, and patient symptoms. Whether additional procedural risk is offset by the physiological and symptomatic improvement that an upgrade can deliver needs to be assessed in a formal manner and is best done with a multi-disciplinary team.^5^

Appropriately designed future studies remain outstanding and are required to help advise clinicians on the optimal timing and circumstances, particularly for those patients with only mild to moderate LV impairment for upgrade.

### Hardware considerations during decision-making

Technical considerations need to be factored into any upgrade/downgrade decision typically focusing on the number and type of existing leads. The downgrade, for example, of CRT-D to CRT-P can more simply be performed with an initial DF-1 lead *in situ* rather than a DF-4 lead. DF-4 leads are often favoured at initial implant as they provide an integrated single connector for the pace-sense function and high voltage components connecting to a single port in an ICD header, therefore, allowing smaller device headers with lower risk of device mis-connections. However, they are less flexible when downgrade is required as they are only compatible with a DF-4 device. Conversely, DF-1 leads, which have multiple ports in the device header with a higher risk of misplacement of high-voltage connectors, can be more flexible for patients with evolving ICD indication and subsequent consideration of downgrade.

With a DF-1 lead, the pace-sense component can simply be connected to a pacemaker generator and the high energy components capped and buried. With a DF-4 lead, either a new IS-1 pacemaker lead needs to be implanted, or if this is not feasible or appropriate based on the patient's risk profile, a replacement device with DF-4 connections may instead be required albeit at significant expense even if the defibrillator components are to be deactivated. Thus, replacement with another CRT-D generator, but turning off VT/VF detections and high-voltage therapies, is a potential approach, rather than lead revision. A range of strategies for this purpose is discussed in the later section dedicated to device downgrades.

Abandoning CIED leads can impact magnetic resonance imaging (MRI) conditionality. Abandoning a lead or a lead component means that MRI-conditionality is lost. However, the 2021 ESC pacing guideline^13^ allows MRI to be performed in patients with abandoned transvenous pacing leads, if no alternative imaging modality is available. Therefore, this imaging modality is not contra-indicated in these patients. No adverse events have been reported from a total of 9 series of 343 abandoned transvenous leads.^61–70^ A further series, which included nine abandoned surgical epicardial leads, also reported no significant adverse events.^71^ However, consideration still needs to be given for mixed-brand systems as well as lead tip heating and device interference. Guidance from a recent cardiac MRI consensus advocates that scanning is preferred at 1.5 T over 3 T for all devices.^72^ It is worth noting, however, despite real-world demonstration of safety that the availability of MRI in many centres is currently limited for patients with abandoned leads and as such, limits the accessibility of this imaging modality.

### Operator experience and need for specialist skills

Upgrading conventional pacemakers or ICDs to CRT systems (BiV or CSP) is in general perceived as ‘complex’. The procedure could be prolonged in duration, associated with increased risk of infections (up to 4.6%)^21^ and might require skills beyond that commonly required for standard *de novo* device implantation. Additional skill requirements might include venoplasty or extraction of leads and the ability to attempt multiple distinct strategies as needed to enable procedural success (ideally in a single sitting).

Complication rates are closely linked to individual and centre implantation volumes.^22–24^ Data from a large national quality assurance programme for pacemakers and CRT-P show that the annual hospital implantation volume is inversely related to complication rates.^73^ These data suggest that CIED procedures (particularly more complex ones) should be performed by operators and centres with sufficient procedural volume and expertise. With more than 90% consensus, it is felt an arbitrary minimum caseload of 20 upgrade procedures/year per centre is an appropriate volume for sufficient experience.

### Pre-procedural steps—importance of procedural planning and contingency planning

Procedural-related complications are higher during upgrade procedures compared with *de novo* implants.^4,5,9,10^ Procedural planning and contingency planning are therefore key to adequately appraise the risk and take the necessary steps to minimize and mitigate these risks.

**Figure 4:**
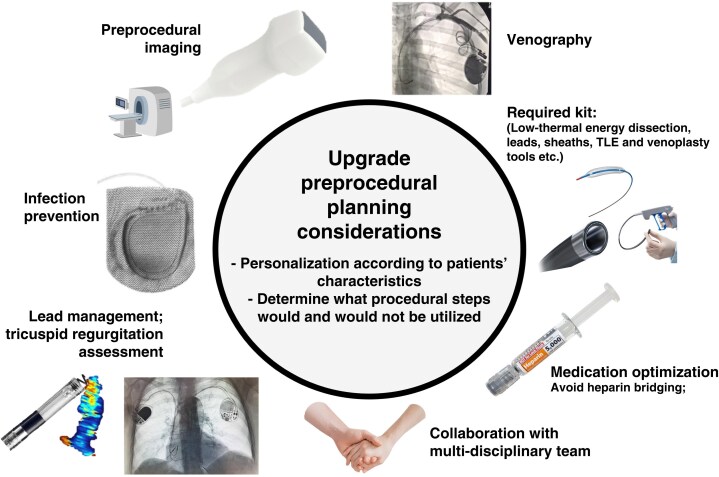
Pre-procedural planning considerations; these include lab set-up, infection prevention, medication optimization, collaborative working approaches, and imaging requirements, including, where possible, a venogram prior to procedure date.

A structured and individually tailored approach to procedural planning allows for the anticipation of procedural challenges, diminishing unpredictability in the procedure, and reducing procedural length and complications. This includes upfront evaluation of venous access, discussion regarding optimal and second-line strategies for lead delivery, optimization of medications including antiplatelets and anticoagulants, consideration of the number of leads crossing the tricuspid valve and the risk of lead-related TR, checks to confirm all required equipment is available and a decision as to what steps would and would not be performed during a case (thus enabling the procedure to ideally be completed in a single sitting)^74^ (*Figure 4*).

Prior to the procedure, the team should plan the procedural steps and how far they will go, balancing net risk against net benefit. This may include setting boundaries as to what approaches would and would not be used for a given patient's procedure i.e. lead extraction or lead tunnelling from a contralateral implant site, and ensuring that appropriate adjunctive teams are available if required e.g. cardiothoracic surgeons and/or anaesthetic teams.

### Value of pre-procedural cardiac imaging

#### Determining the need for upgrade—assessment of left ventricular function

Two-dimensional echocardiography is the first imaging technique to evaluate LVEF in patients who may be candidates for an upgrade to CRT. Assessment of myocardial deformation patterns by strain imaging or the use of three-dimensional (3D) echocardiography might provide additional useful information personalizing the upgrade approach.^75–78^ The presence of apical rocking, septal flash, and use of speckle tracking can provide a useful assessment as to the presence of dysynchrony.^79,80^ Additionally, cardiac MRI may be useful to evaluate myocardial scar and arrhythmic risk in patients with a potential ICD indication.

#### Assessment of tricuspid regurgitation

Echocardiography can identify patients with significant TR. In such patients, additional leads across the tricuspid valve may worsen TR and cause right-sided HF. However, it remains unclear for patients who already have severe TR whether they will experience a deterioration in symptoms or valve function if additional leads are placed across already severely affected valves.

There is equipoise regarding whether bulkier and stiffer ICD leads may impact greater upon TR than smaller pacing leads or whether any association seen relates to lead lie, trajectory, and its location rather than size.^81–83^

Where worsening of TR is of significant concern, there are potential alternative approaches that avoid additional leads across the tricuspid valve. For pacing, this could include epicardial leads, coronary sinus (CS) leads, and His pacing (on the atrial side of the tricuspid valve) and even leadless pacemakers.^84–86^ Leadless pacemakers (potentially related to implant technique and location of deployment) can, however, in some cases, worsen TR from baseline.^87^ For defibrillation, this could include S-ICD (Boston Scientific, USA), EV-ICD (Aurora EV-ICD™, Medtronic, USA), epicardial ICD leads or standalone ICD coils placed in alternate locations.^85^ Standalone coils are rarely used in current practice, however, when used, these coils can be placed in the azygos vein or CS connected to the RV port or a DF-1 ICD coupled with an antero-lateral subcutaneous ‘SQ’ array + epicardial/CS pacing lead (connected to IS-1 RV port) or placement of an ICD lead in the middle cardiac vein. Currently, the only available products for such purposes include the 6996SQ subcutaneous coil (Medtronic, USA) and the Transvene 6937 unipolar SVC coil (Medtronic, USA).

An alternative approach is to consider the extraction of redundant leads to avoid inactive leads crossing the valve. It is unclear whether this mitigates against increased TR risk.

### Additional utilization of imaging

#### Cardiac computerized tomography

A multi-modality approach based on 3D imaging techniques may provide useful pre-procedural information. Cardiac computed tomography angiography might offer information on venous patency prior to a planned upgrade procedure. It is unclear clinically whether this is superior for procedural planning than a peripheral venogram.

Computed tomography (CT) angiography can provide additional detailed assessment of coronary anatomy (which may prompt further coronary assessment in the context of LV deterioration if significant coronary artery disease is seen). Evaluation of coronary venous branch anatomy can also be performed to assess upfront CS anatomy; however, it requires X-ray radiation and iodinated contrast.^88^

#### Cardiac magnetic resonance imaging

Cardiac MRI provides detailed information about scar, ventricular function, and degree of segmental ventricular contraction, but requires longer scanning times and may be limited by already abandoned leads or previous concerns regarding device incompatibility, which limits access to MRI at many centres.

Randomized trials have not systematically demonstrated that the guidance of LV lead implantation based on MRI imaging to localize myocardial scar is superior to standard implant practices; however, a recent RCT did show improvement in its secondary endpoint of LVESV reduction when MRI-guided lead position was used in CRT recipients.^88–91^ The absence of a suitable CS vein over viable myocardium may discourage LV lead placement and potentially favour CSP placement or a leadless LV pacing approach.^92–94^

MRI may be useful in patients with PICM, where the absence of scar may help inform arrhythmic risk. Compelling RCT data, however, is lacking, and as such, the use of MRI to determine the need for a defibrillator based on scar pattern is an area of uncertainty.^95,96^

#### Upfront superior vascular anatomy assessment: the venogram

Peripheral venography via a peripheral vein prior to skin incision can be very useful in order to plan any upgrade procedure. This is often the preferred approach for procedural planning to characterize venous anatomy rather than CT or other imaging modalities.

It is often advantageous, where possible, to perform venography in advance of the planned procedure date in patients, particularly in whom there is a high suspicion of venous occlusion (i.e. more than 3 leads *in situ* or the presence of visual venous collateralization on the chest wall).

Performing the venogram in advance, and discovery of significant stenoses enables upfront discussion of available options with the patient and planning of the procedure i.e. alternative venous approaches and whether proceeding with extraction is likely to be required or not. While this is ideal, it may not always be possible to arrange venography in advance of an upgrade procedure, but it should be done at the very least prior to skin incision. Contrast venography can identify subclavian or innominate vein stenosis/occlusions as well as highlight congenital abnormalities (e.g. persistent left superior vena cava^97^ information that should be known before starting the procedure).^98^

When attempting to gain venous access, it may be useful to use a caudal tilt to guide puncture.^98^ It is also worth noting that peripheral venography may overestimate the presence of occlusions and more selective dye injection via a dilator within the vein can reveal residual flow through the lesion in approximately two-thirds of cases where initial venography has suggested occlusion supporting the use of proximal venography i.e. using the axillary vein in addition to peripheral venography in such case.^99^ These significant venous narrowings (including those that are near-complete) may then be crossed using hydrophilic wires and venoplasty performed as needed.^100^

Ultrasound imaging is sometimes utilized to assess vein location, patency, and guide vascular access; however, this approach is limited in that it cannot demonstrate more central occlusions.^101,102^ As such, it is regarded as an inferior choice to contrast venography for pre-procedural planning and should not be used for this purpose.^103^

### Anaesthetic considerations

Local anaesthesia (LA) with or without sedation is often preferred for device procedures, including upgrades. This approach generally has fewer cardiovascular and respiratory risks making it safer for high-risk patients. This is particularly true for patients with emphysematous lung disease, where already hyperinflated lungs can be further inflated with significantly reduced margins between the lung surface and the axillary vein giving a higher pneumothorax risk while obtaining vascular access. Recovery times are shorter, more readily enabling cost-effective day case procedures. However, patients undergoing long or complex cases may struggle to remain still and, in these scenarios, moderate sedation or general anaesthesia (GA) allows for precise procedural control without compromising patient comfort. It additionally allows complex and potentially painful procedures to be completed in a single sitting, including the use of tunnelling and extraction where needed.

Anaesthetic provision should be assessed alongside all the other pre-procedural planning steps to ensure that a case can be completed as planned ideally in one sitting.^104^ This may mean for a patient with a widely patent venous system that GA is not required, but for a patient with a stenosis where extraction or contralateral tunnelling may be necessary, GA should be used (or at least on standby) from the outset. In a high-risk patient where the extent of procedural complexity to facilitate venous access has been set at venoplasty alone, LA or GA are both potential options based on operator and patient preference, and ease of availability of anaesthesia. If required, a defibrillation test (DFT) may also influence the upfront choice of sedation or general anaesthesia, as many may find it preferable to perform DFTs under general anaesthesia or deep sedation.

### Medication considerations

#### Anticoagulation and antiplatelets

Anticoagulant use is associated with a high risk of haematoma formation, as is antiplatelet therapy.

The BRUISE-CONTROL 1 study showed increased haematoma formation with heparin bridging therapy in those patients taking warfarin undergoing ICD or pacemaker implant compared to uninterrupted warfarin use.^105^ The BRUISE-CONTROL 2 study did not show any difference in the risk of haematoma with continuous uninterrupted direct oral anticoagulant (DOAC) use for device procedures.^106^ Limitations with this study though are the enrolment only of patients undergoing *de novo* procedures and the occurrence of few events.

In a sub-analysis of the WRAP-IT trial (*n* = 6800), multi-variable modelling identified prior CIED procedures and lead revision as being associated with increased haematoma risk.^107^ Similarly, in the REPLACE registry, the documented rates of haematoma for device upgrades or lead revision procedures were 3.5% and 4.3%, with the highest incidence in CRT device upgrade procedures.^40^ However, in a large single-centre registry of 2100 patients, device upgrade was not associated with a higher risk of haematoma formation.^108^ In the WRAP-IT trial, those patients with an acute haematoma were observed to have a >11-fold risk in the development of CIED infection during 3 years of follow-up. This risk was significantly reduced by the use of an antibiotic envelope.^109^

In patients where the risk of bleeding is deemed to outweigh the risk of stroke or embolic complication [e.g. submuscular pocket formation, bleeding diathesis, or transvenous lead extraction (TLE)] it is advised to hold anticoagulation for 24–48 h prior to device upgrade procedures and resume after 24–48 h. For patients on dual antiplatelet therapy (DAPT), aspirin can be continued, but it is advised to hold the second agent; 3 days for ticagrelor, 5 days for clopidogrel, and 7 days for prasugrel beforehand. The second antiplatelet agent can be resumed after 24–48 h. Where DAPT interruption is not feasible (such as recent stent placement) careful risk assessment and individualized planning are advised.

Procedures can frequently be performed on uninterrupted single agent low-dose aspirin.^110^

### Equipment and lab set-up considerations

*Table 5* provides a summary of the required and desirable equipment and lab set up for device upgrades.

**Table 5:** Required and desirable equipment and lab set up for device upgrades

Equipment and lab set-up for device upgrades	
Essential	Access to general anaesthesia (either as an upfront option, or if not used upfront, should be immediately available in case of extraction and may be useful in other complex, long duration procedures or those that involve lead tunnelling)^111^ Venography tools (peripheral and coronary sinus) and fluoroscopy equipment with recording capacity for venography Hydrophilic guide catheters, hydrophilic wires, hydrophilic sheaths of sufficient length, and stiff wires If transvenous lead extraction is to be performed, general anaesthesia, invasive monitoring, lead extraction tools,^56^ and availability of immediate surgical backup^48^
Desirable or may be useful	Venoplasty tools^52^; tunnelling tools, micropuncture catheters, non-compliant peripheral balloons Low-thermal energy dissection devices [for example, pulse electron avalanche knife (PEAK) PlasmaBlade™ (Medtronic, USA), or PhotonBlade (Invuity, USA)] Extraction centres should be prepared for all possibilities including the need for femoral extraction techniques^53^ EP recording system with 12-lead ECG in order to assess intracardiac electrograms and QRS width and morphology (when a conduction system pacing approach is being considered)^54,55^ Material for biventricular implant, His bundle pacing, and left bundle branch pacing; be prepared for a change in approach to or HOT/LOT-CRT (his or left bundle branch-optimized cardiac resynchronization therapy) strategy

**Table of Advice:** General considerations

General considerations	Strength
**Advised to do**	
In patients who have received a conventional pacemaker or an ICD and who develop symptomatic HF with LVEF ≤ 35% despite OMT and who have a significant (>20%) proportion of RV pacing, upgrade to CRT is advised^16–19,22,27,57^	>1 moderate quality RCT or Meta-analysis of moderate quality RCT
In patients who have received a conventional pacemaker and who develop an ICD indication, ICD upgrade is advised^27^	>1 moderate quality RCT or Meta-analysis of moderate quality RCT
An integrated care approach is advised in pacemaker and ICD patients when considering upgrades and downgrades to ensure a patient-centred approach with patient involvement and shared decision-making	>90% of writing group agree
When HFrEF is detected in the presence of high RV pacing burden, it is advised to discuss an upgrade strategy utilizing a multi-disciplinary team approach, including consideration of prognosis and risk/benefit analysis. Discussion should include discussion of upgrade to CRT with or without ICD capability	>90% of writing group agree
It is advised that device upgrade procedures are performed by experienced operators in experienced centres (at least 20 upgrade procedures/year)	>90% of writing group agree
**May be appropriate to do**	
In patients who have received a conventional pacemaker or an ICD, and who subsequently develop symptomatic HF with either LVEF 36–40%, or a significant (∼ > 10%) decrease in LVEF from baseline despite OMT, and who have a significant (>20%) proportion of RV pacing, it may be appropriate to upgrade to CRT^16,20^	High quality, large observational studies
**Areas of uncertainty**	
It is uncertain whether patients who suffer from PICM alone, with no other substrate identified for LV impairment, benefit from upgrade to CRT plus a defibrillator, or rather just upgrade to CRT strategies	>90% of writing group agree

**Table:** 

Patient selection and assessment for upgrade indications and generic considerations	Strength
**Advised to do**	
At all planned device follow-up visits (by physicians/cardiac physiologists/technicians/device nurses), assessment as to whether a patient has the most appropriate device implanted is advised. This should include review of symptoms, hospitalizations, and pacemaker-derived data review. This assessment may trigger further initial investigations with echocardiography and/or NT-Pro-BNP	>90% of writing group agree
When patients with a ventricular pacing burden >20% are approaching ERI (<12 months), evaluation of symptoms of heart failure and left ventricular function is advised	>90% of writing group agree
When LVEF deterioration is confirmed, investigation of secondary causes, including arrhythmias, coronary atherosclerosis, lack of GDMT etc. and involvement of HF specialists or invasive cardiologists as needed is advised	>90% of writing group agree
A shared decision-making process is crucial before the upgrade procedure considering patients’ preferences and taking into consideration frailty and comorbidity burden. The multi-disciplinary team may play a key role in this	>90% of writing group agree
When lead extraction is being considered, it is advised to involve a multi-disciplinary team including the CIED specialist, extraction specialist, anaesthesiologists, cardiac surgeons, geriatricians, and infectious diseases specialists where appropriate	>90% of writing group agree
**May be appropriate to do**	
It may be appropriate to perform device upgrade at the time patient symptoms or LV function deterioration is noted and not defer till time of elective generator replacement^27,112^	>1 moderate quality RCT or Meta-analysis of moderate quality RCT

**Table:** 

Pre-procedural considerations	Strength
**Advised to do**	
Before an upgrade procedure, it is advised that procedural steps and strategy are planned, including what steps (for example venous access strategies or extraction) would and would not be used for a given patient balancing net risk against net benefit	>90% of writing group agree
Two-dimensional echocardiography is advised as the primary imaging technique to evaluate patients who may be candidates for an upgrade to CRT	>90% of writing group agree
It is advisable to have a recent echocardiogram before replacing a device generator, to assess LVEF in those with ventricular pacing >20% and/or with heart failure symptoms (preferably done within 12 months of the proposed generator replacement)	>90% of writing group agree
Before a generator replacement, it is advised to evaluate patient's condition for signs and symptoms of heart failure and to check the device for ventricular pacing burden as well as atrial and ventricular arrhythmias	>90% of writing group agree
Where the risk of bleeding significantly outweighs the risk of stroke or embolic complication, it is advised to hold anticoagulation for 24–48 h prior to device upgrade procedures and resume after 24–48 h.	>90% of writing group agree
Where risk of bleeding outweighs the risk of coronary complications (i.e. stent thrombosis), it is advised to withhold DAPT for an appropriate duration prior to procedure (3 days for ticagrelor, 5 days for clopidogrel, and 7 days for prasugrel) whilst continuing on with aspirin and resume the second antiplatelet agent after 24–48 h	>90% of writing group agree
Venography should be performed before skin incision. where possible, it is advised to undertake this in advance of a planned upgrade procedure date to enable procedural planning	>90% of writing group agree

## Vascular superior access considerations

### Vascular access issues

Venous obstruction in patients with CIEDs is not uncommon. Lead-related venous obstruction has been categorized according to the degree of maximal narrowing, ranging from mild in up to 40% of patients^113,114^ to severe or total occlusion in 3–9% of patients.^115–117^

Venous abnormalities that occur soon after transvenous device insertion are usually related to venous thrombosis without a coexisting stenosis.^118^ However, venous thrombosis that occurs later than 1 year after implantation likely stems from a stenosis caused by organization of a previous thrombus.^119,120^ The pathogenesis of venous thrombosis after implantation of a transvenous CIED is multi-factorial. Ligation of the vein used for access (cut-down approach) can cause a central extension of thrombus from the cephalic vein to proximal large veins.^121^ The development of collateral circulation may cause further slowing of blood flow in the affected vein and create a prothrombotic state.^122^ Endothelial trauma caused by pacemaker leads may also cause an inflammatory response of the surrounding vessel wall with subsequent scarring and subsequent fibrosis.^123^ More recently, multi-level lead-related venous stenosis/occlusion (MLVSO) has been proposed as a better index of the severity of global venous obstruction (rather than the degree of vein narrowing at only a single point), and this has been explored in a large cohort of 3002 patients.^124^ The number of leads in the heart, presence of CS leads, leads on both sides of the chest, and a previous device upgrade or downgrade with lead abandonment were the strongest predictors of significant MLVSO.

**Figure 5:**
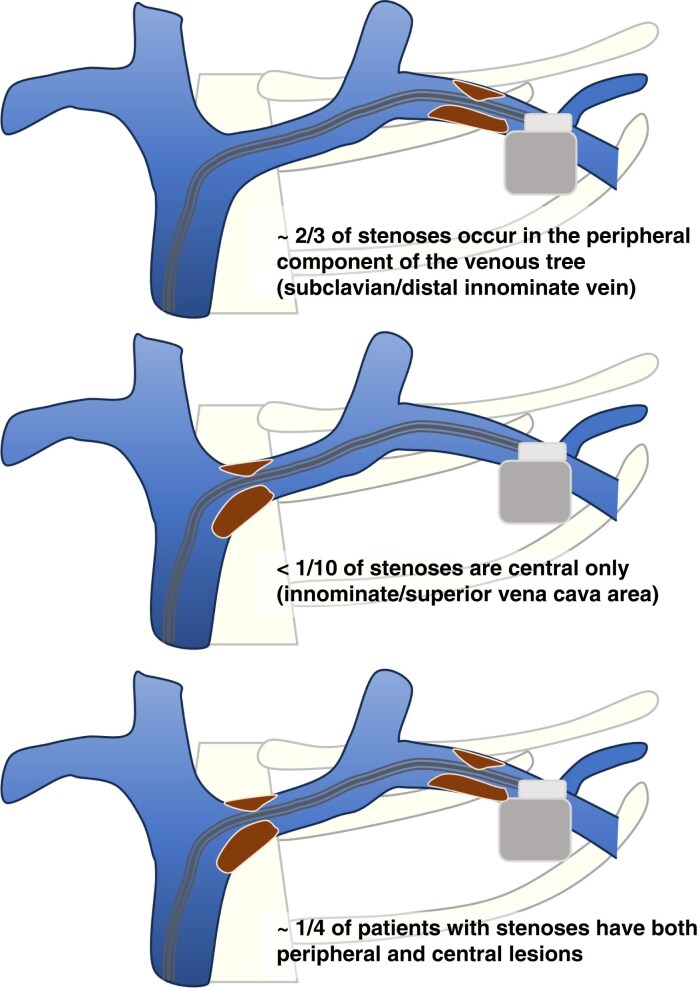
Venous stenosis locations and their prevalence. The majority of venous stenoses occurs peripherally in the venous tree.^114,125^

Venous stenosis/occlusion is usually asymptomatic but may become clinically important in the event of a device upgrade. The majority (∼2/3) of significant stenoses or occlusions are located in the peripheral component of the venous tree (subclavian/distal innominate), while a minority are central only (innominate/superior vena cava area (∼17%),^100^ whilst ∼25% of patients with venous stenoses involve both peripheral and central locations (*Figure 5*).^100,114,125^ Typically, venous occlusions have associated collateral veins; however, these are typically small and tortuous and therefore cannot be used to place a new lead.

### Techniques to manage venous stenoses/obstruction

**Figure 6:**
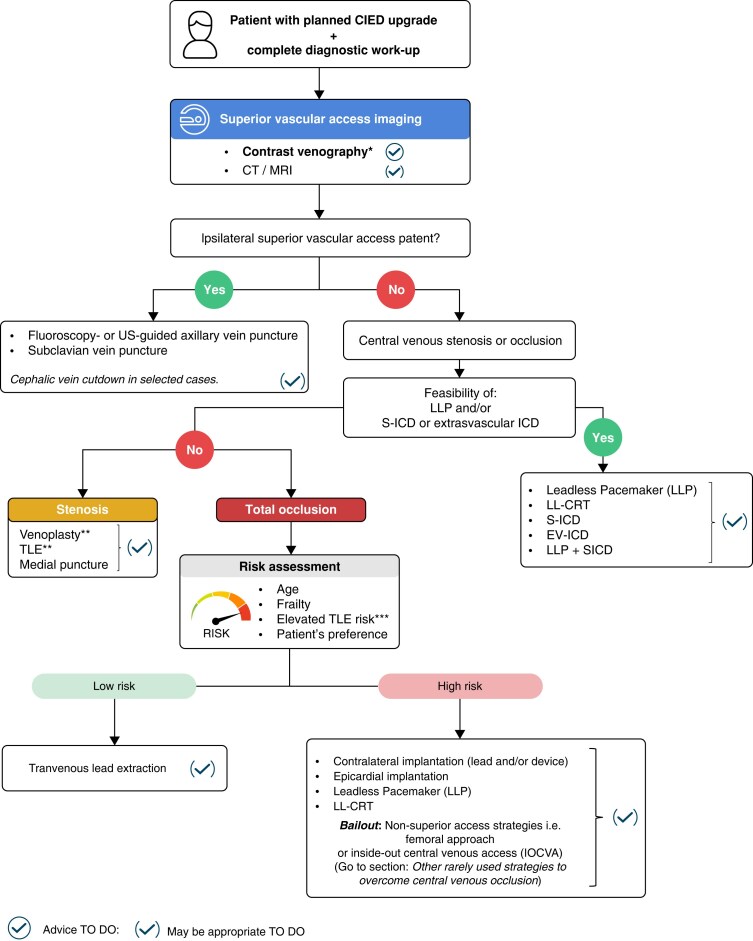
Decision tree for optimal superior vascular access in planned CIED upgrade procedures. *Contrast venography = preferred imaging to plan an upgrade procedure; **Venoplasty or TLE depending on the operator and/or centre experience and informed consent with the patient; ***Risk factors for TLE: (age, low BMI, advanced heart failure, severe LV dysfunction, renal dysfunction, number of implanted lead, dual coil ICD lead, diabetes). Abb.: CT, computed tomography; LLP, Leadless Pacemaker; MR, magnetic resonance; S-ICD, subcutaneous ICD; TLE, transvenous lead extraction; US, ultrasound.

Multiple techniques are available for patients with venous stenoses or obstruction and a planned CIED upgrade with pre-existing leads. Superior vascular access (i.e. axillary, subclavian, and cephalic) strategies are preferred over non-superior access (i.e. femoral) strategies, which may, however, be helpful in bailout situations. Such situations are becoming less frequently required due to adoption of more novel alternative approaches (i.e. leadless pacing).^86^ The chosen strategy to overcome superior vascular issues depends on multiple factors such as operator and centre experience, patient-specific risk factors, venous anatomy/location, and size of the venous obstruction, and the patient's preference (*Figure 6*).

*Table 6* summarizes the current vascular access options and their advantages and disadvantages, which are described in detail in the following sections.

**Table 6:** Summary of strategies to obtain vascular access in patients with a planned CIED upgrade procedure

Vascular Access options	Advantage	Disadvantage	For whom?
**Superior vascular access options**			
Medial subclavian vein puncture^126–128^	Ipsilateral vascular access Preservation of contralateral vascular access Relatively easy technique	Increase in risk of pneumothorax and haemothorax Risk of long-term lead damage (subclavian crush) impacting on lead performance Success rate variable depending on the occlusion (length, complete occlusion)	Favourable anatomy of the venous stenosis or occlusion (puncture distally) TLE and venoplasty not appropriate or available
‘Wire under the insulation’ technique^129^ (figure demonstrating technique included in reference)	Preserves vascular access Avoids repeated venous cannulation Minimally invasive	Highly challenging in patients with extensive venous thromboses or occlusion (therefore less useful in leads with longer dwell time) Success rate variable with risk of lead damage	Patients with limited vascular access Relatively short lead dwell time Patients at high risk of complication from repeat venous cannulation (i.e. those with COPD or frailty)
Serial dilation or venous venoplasty^85,86^	Ipsilateral vascular access Preservation of contralateral venous access	Procedure duration potentially increased Potential damage to remaining leads Multi-disciplinary approach occasionally needed (interventional radiology support) but mostly can be done by cardiologist alone Training/experience required	All CIED upgrades with a suitable pre-operative venogram Upgrade in patients with <4 leads on one side and <5 through the SVC
Transvenous lead extraction (TLE)^130–132^	Ipsilateral vascular access Preservation of contralateral venous access Lead management in young patients i.e. minimises total number of leads MRI conditional system	Procedure duration potentially increased Additional risks including SVC tear, haemothorax, tricuspid valve damage, tamponade, death. Collateral damage (TLE of all leads instead of only a redundant lead due to inter-lead fibrosis), possible extraction of functional leads Availability of expertise and centre capability Femoral access requirement Cardiothoracic surgery backup generally required Availability of general anaesthesia	Young age and long-term need for functional CIED therapy and desire to avoid an abandoned lead Upgrade in patients with venous occlusion + ≥1 dysfunctional lead or ≥1 functional lead to be replaced Single (non-ICD) lead for extraction, lead dwell time <10 years, no coronary sinus lead (risk of inadvertent extraction along with other leads) [lower risk] MRI conditional system required
Contralateral approach^133^ Contralateral implantation of a new system Contralateral lead implantation plus tunnelling to initial site	No complications associated with venous intervention or lead extraction Increased procedure duration Increased risk	Contralateral approach with both vascular access sides used Risk of bilateral subclavian vein occlusion Increased number of leads through the SVC (potentially higher risk of SVC syndrome) Future lead revisions are more challenging Potential risk of lead-lead abrasion or interaction Further compromise of venous access If total system extraction required secondary to infection, future device implantation options may be limited due to infection involving both right- and left-sided access sites True MRI-conditionality lost contralateral tunnelling carries risk of cutaneous lead perforation	Older patients Frail patients High-risk associated with TLE (low body-mass index (BMI) < 25 kg/m^2^, severe LV dysfunction, renal dysfunction, complete venous occlusion, leads >10 y/o, high-risk leads)^134^ Low total number of leads (right and left; ≤4, ≤1 ICD lead in total) Non-ICD lead (for contralateral tunnelling)
Epicardial/surgical lead implantation^135–137^	Lead can be readily placed if patient undergoing cardiac surgery for alternate reason	Thoracotomy Additional risks of surgery Additional cost Suboptimal lead performance Difficult removal or revision Non-MRI conditional system	CRT upgrade after failed endocardial lead positioning and failed CSP For patients undergoing surgery for other reasons
Utilization of a non-transvenous approach (e.g. LLP, S-ICD, EV-ICD, WISE CRT)^138–156^	No lead associated problems in LLP Risk of CIED related endocarditis is lower	Only available for selected patients depending on the planned up- or downgrade procedure Unclear future management of LLPs in patients <40 y/o (limited long-term data available at the current time) Additional cost CSP LLP not readily available at this time Only a limited option in CRT	Patients at a high risk of CIED infection (e.g. haemodialysis, prior CIED infection, immunosuppression) ATP not required (S-ICD) Bilateral venous occlusion Patients at high risk for TLE
**Other, rarely used strategies to overcome central venous occlusion (non-superior access)**			
Inside-out central venous access (IOCVA)^157^	Ipsilateral vascular access Classical pectoral placement of pacing and/ or defibrillation leads Applicable for chronic total SVC and subclavian vein occlusions	Higher potential risk of pneumothorax and haemothorax Limitations in patients with scarred or calcified subcutaneous tissue No experience with lead revision or TLE Unknown risk to existing leads Complex intervention for experienced operators	Bailout strategy for very few patients
Femoral (or *trans*-iliac) approach with abdominal or thigh pulse generator placement^158–162^	Pacing option in patients with central venous occlusion (predominately in the era prior to LLP)	Unstable leads Risk of lead dislodgement, especially atrial lead Additional subcutaneous lead in defibrillator systems to overcome the abdominal pulse generator location Risk of deep vein thrombosis and pulmonary embolism Increased risk of procedural complications (infection, lead fracture, intestinal/vascular injury)	Bailout strategy for very few patients, primarily for implantation of a new CIED in patients with complete central venous occlusion (particularly relevant now if LLP is not available/suitable)

Options outlined should be discussed with the patient, taking into account, where appropriate, their views relating to procedural risks, for example when a patient desires to seek an alternative to a transvenous lead extraction.

Abbreviations: ATP, anti-tachycardia pacing; CIED, cardiac implantable electronic device; CRT, cardiac resynchronization therapy; CSP, conduction system pacing; IOCVA, inside-out central access; LLP, leadless pacing; LVEF, left ventricular ejection fraction; MRI, magnetic resonance imaging; S-ICD, subcutaneous ICD; SVC, superior vena cava; TLE, transvenous lead extraction.

### Vascular superior access considerations

#### Preparation

Information on how previous leads have been implanted is important. If a cephalic cut-down technique has previously been used,^126^ the cephalic vein cannot be utilized again as a method of access. If axillary or subclavian puncture has been used for the original implanted leads, the cephalic approach may be possible,^163^ although fibrosis in the pocket area may make it difficult to cleanly dissect the tissues and expose the vein.

It is essential to obtain good quality ipsilateral contrast venography of the chosen vein for puncture to examine the anatomical route of the distal part as well as to assess the patency of the vein from where the original leads enter the vessel, all the way to the superior vena cava.^114^ If collaterals are observed, it is often a sign of at least partial stenosis, but not necessarily total occlusion. It might be possible to pass guidewires through such a part of a vein.

If an occlusion is observed and a guidewire does not pass along the lead, it can sometimes be helpful to visualize the extent of the occluded section by venography proximal to the occluded site with a guiding catheter via femoral access through the right atrium and superior vena cava.

#### Puncture

The site of puncture and orientation of the needle can be guided by the contrast filling of the distal part of the vein or more proximally by the route of the previously implanted leads.^164^ The diameter of the needle used for puncture is often 18 G but newer Micropuncture® (Cook Medical, USA) or Merit MAK™ Mini Access Kit (Merit Medical, USA) come with a thinner 21G needle, and a 4 or 5 Fr sheath with a nitinol guidewire with a straight, yet flexible, tip may be useful. No formal studies have evaluated these against standard equipment in CIED implantation, but the approach has been suggested to be advantageous in non-cardiac venous access procedures.^165^ It is important to avoid damage to the existing leads by evaluating the course of the needle as well as its interaction with the lead under fluoroscopic guidance. A thinner needle might reduce that risk, in addition to reducing the risk from inadvertent arterial puncture.

Puncture should preferentially be performed in an extra-thoracic location either in the axillary vein or the distal subclavian portion (overlying the first rib), if there are no signs of occlusion.

A medial subclavian vein puncture (where required) involves puncturing the subclavian vein closer to the sternoclavicular junction, which can bypass a stenotic/occluded area. However, this carries an increased risk of pneumothorax, arterial puncture and haematoma, rarely arterio-venous fistula, transient phrenic nerve palsy due to LA, thoracic duct injury, and potentially greater risk of future lead dysfunction. This approach should be reserved as a strategy when first-line options are not successful.^126,127^

Standard *de novo* vascular access for implantation can be guided by ultrasound.^101,102,166^ However, in upgrade procedures, it has not been systematically evaluated, and fluoroscopy frequently remains the method of choice for guiding the puncture as ultrasound cannot always visualize proximal stenoses, which may preclude successful access to the heart. Ultrasound-guided puncture can, however, guide and aid puncture into the axillary vein upstream to any stenosis at low risk to the pre-existing leads. Therefore, for some operators, a hybrid approach using venography and ultrasound has a possible role.

### Access through an occluded vessel

If venography identifies a possible occlusion, a hydrophilic guidewire (for example, a Whisper wire; Abbott, USA, or the Laureate wire, Merit Medical, USA, or the wire from the Merit MAK™ Mini Access Kit, Merit Medical, USA) is usually required for gaining access beyond the stenotic section.^99^ It is often not possible to confirm a total occlusion (where a guidewire cannot be passed), just based on peripheral venography alone. In case of a total occlusion or near-total occlusion, wires and techniques used for crossing occluded coronary arteries can also be used to cross chronically occluded veins.^99,167^ The principle is to use wires that are increasingly more aggressive and stiffer. Once a wire has passed the occluded portion and is safely in the right atrium or the inferior vena cava, addition of microcatheters allows gradual upsizing to larger and stiffer guidewires and finally insertion of a large enough introducer sheath.

Alternatively, a hydrophilic angled braided guide catheter (for example the KA2 guiding catheter, Merit Medical, USA) can be utilized to provide direction and support for wire passage. On occasion, advancing the catheter with puffs of contrast without a wire can be more effective than blind wire advancement. Once a wire has crossed the lesion/s and is safely in the inferior vena cava, serial dilatation or venoplasty may be performed.

### Serial dilatation

Serial dilatation involves progressively enlarging the vein through the vascular entry point using a series of dilators of increasing diameter. This method is employed after the initial vessel puncture and guidewire insertion. The primary goal is to create a sufficiently large and stable access route to facilitate the introduction of the CIED leads through the gradual and controlled expansion of the vessel. Depending on the severity of stenosis and the type of procedure, a range from standard sheaths to specific dilator kits can be used. Using a super-stiff 0.035″ wire e.g. Supra-Core™ (Abbott, USA) or Amplatz Extra Stiff™ (Cook Medical, USA), is usually necessary to provide support, especially when navigating an angulated route. Due to the stiffness of the larger-sized dilators, this technique is usually restricted to straight distal stenotic segments. The access created through serial dilatation is often temporary, necessitating prompt advancement of the necessary equipment. Complications of this approach very rarely include vessel perforation, excessive bleeding, and haematoma formation.

### Balloon venoplasty

**Figure 7:**
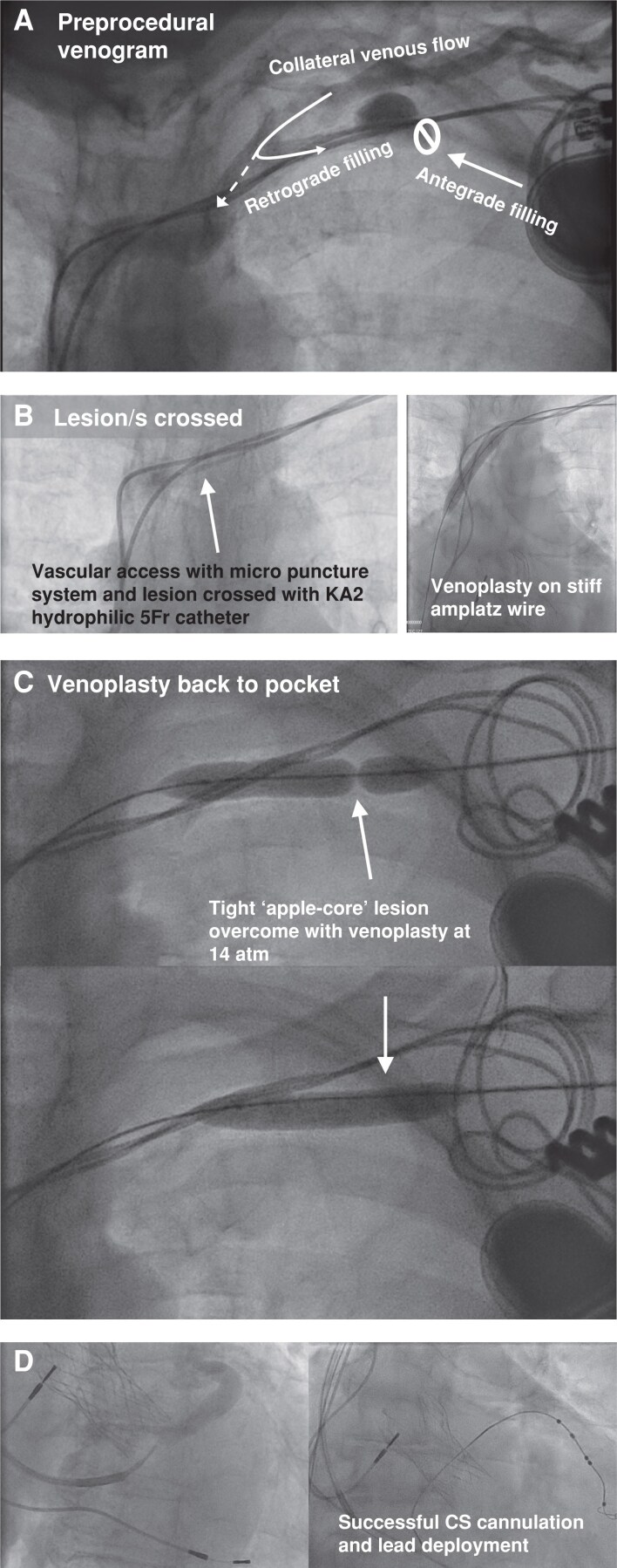
Venoplasty case example during an upgrade to CRT-P. (*A*) Pre-procedure venogram shows a venous stenosis interrupting antegrade flow, the presence of collateral vessels, then backfill up to the point of stenosis. (*B*) A KA2 hydrophilic guide catheter is used to cross the stenoses. (*C*) Venoplasty is then performed over a stiff Amplatz wire. A tight ‘applecore’ lesion is seen at the site of stenosis on the initial venogram, which is overcome by balloon inflation to 14 atm/14.18 bar. (*D*) This approach permits sufficient venous access to successfully deploy an LV lead.

Stenotic veins will hinder access to the heart for pacing leads and also the manoeuvrability of guiding catheters for either BiV pacing or CSP lead placement. In such cases, venoplasty can be performed.^100,167,168^ Venoplasty can be done safely and efficiently by a device implanter in a single sitting. As most stenoses are fibrotic, they may be prone to elastic recoil after venoplasty has been performed and as such it may be preferential to perform venoplasty and the device upgrade in a single sitting. The procedure is typically performed utilizing a non-compliant balloon (typically 6–10 mm diameter by 40–60 mm length) over a stiff guidewire. In some cases, pre-dilatation with a smaller balloon-catheter is required to facilitate access for larger guidewires and thus larger balloons. Typically, balloon dilatation is performed from the RA-SVC junction all the way back to the access site in the pocket with inflations to the rated burst pressure of the balloon (for example 14 atm or 14.19 bar for the Boston Mustang 10 mm × 40 mm balloon and 12 atm or 12.16 bar for the Abbott Armada balloon).^100^  *Figure 7* shows a case example. Venous stenting in such patients is not a favoured approach without performing lead extraction first, due to the risk of ‘jailing’ the lead and the potential risk of lead damage.^169,170^

### Infrequently used methods

- PowerWire (Baylis Medical, Canada) is a specialized tool designed to facilitate vascular access, particularly in challenging cases where traditional methods are insufficient. This device utilizes radio frequency (RF) energy to traverse occluded or stenosed veins, providing a pathway for subsequent device implantation procedures. There are only limited published data regarding efficacy and safety of this approach.^171^
- The inside-out access approach is a minimally invasive vascular access technique. In this method, a trans-septal needle within a long sheath is advanced from the femoral vein up to the most central point of a stenosis. From here, the vein can be punctured from within, creating a controlled exit to a predefined supra- or infra-clavicular point. Following this, a wire is advanced through the trans-septal needle inside-out, which is then used as a rail to introduce a sheath on to secure central venous access. Experience of this technique being used for CIED implantation is very limited.^157^

### Contralateral new lead implantation and tunnelling/contralateral new system implant and abandonment of original system

Implantation of an entirely new system on the contralateral side can be problematic since the most frequent upgrade procedure is from a dual-chamber pacemaker to a CRT system, which may lead to 5 leads in the SVC with an increased risk of superior vena cava obstruction/venous thoracic outlet syndrome. If fewer leads are involved, this can more readily be considered. However, even fewer than 5 leads in some patients may increase the risk of SVC obstruction, and therefore, this decision should be individualized.

Implantation of a single lead for system upgrade on the contralateral side with tunnelling to the original side represents an alternate although less frequently adopted option (*Figure 9*). This approach appears safe; however, it should be noted that the data documenting the longer-term performance of tunnelled leads is limited to three registries (*n* = 72) over median follow-up of 2–3 years.^133,172^ Utilization of the contralateral vasculature should be carefully assessed as an option in the decision-making process as doing so may compromise future options for re-implantation or revision in the event of infection, which would then affect both right- and left-sided vascular access sites.

**Figure 8:**
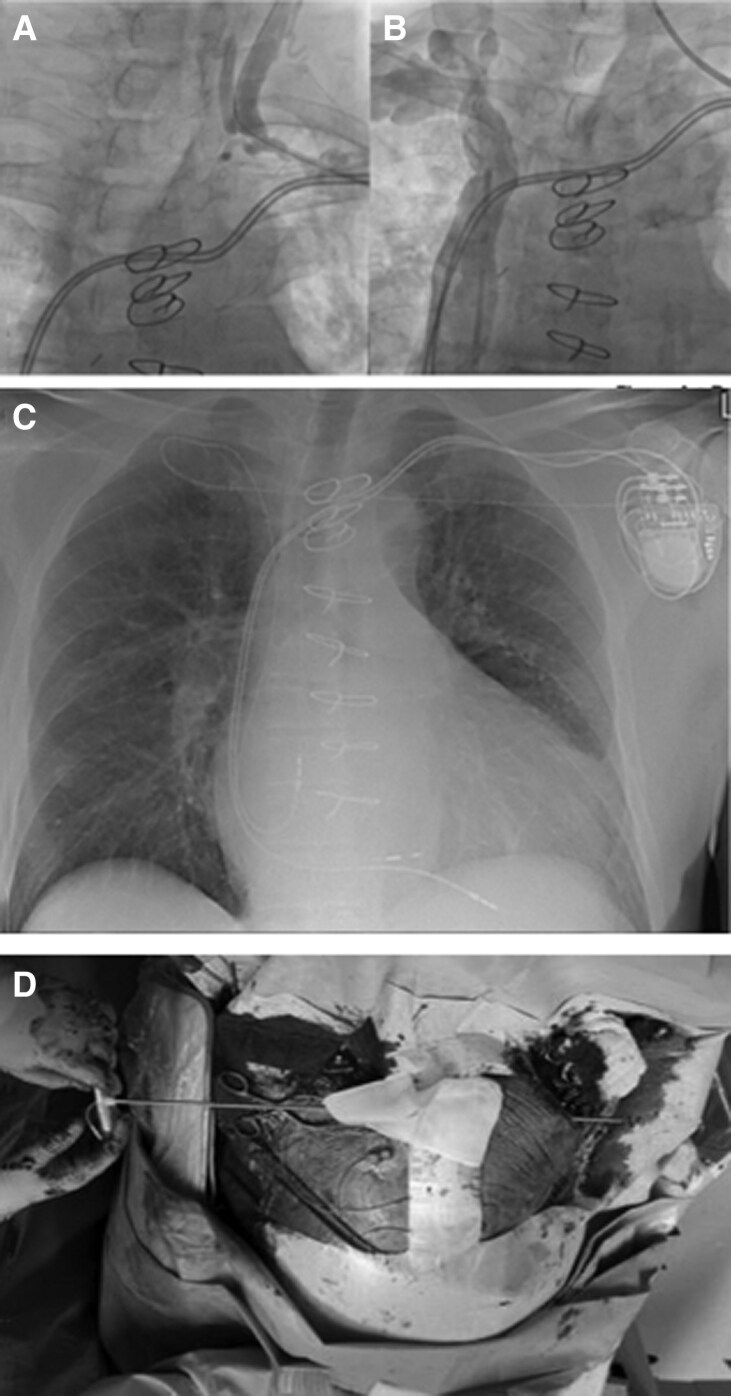
Upgrade of a dual-chamber pacemaker to a left bundle branch area pacing triple lead system in a patient with subclavian vein occlusion. (*A* and *B*) Venography from the left subclavian vein and the SVC by a sheath introduced from the femoral vein reveals a very long and complete total occlusion of the left subclavian vein. All attempts of reopening or passing the occlusion through collaterals were unsuccessful. (*C*)Thoracic X-ray after implantation of a left bundle branch pacing lead from the right subclavian vein. The lead is fixed with a sleeve on the right side, then tunnelled straight to the left-sided device pocket. (*D*) Operation situs. The tunnelling device (Codman disposable catheter passer), with a tapered nylon tip, has been pushed suprasternal to the pacemaker pocket on the left side. After the grab handle (white) has been removed, the IS1 connector tip is attached to the white inner plastic by a suture, and the olive tip with the plastic wire inside the tunnelling device can be pulled out towards the device pocket. Finally, the metal tube is removed towards the device pocket side.

The new contralaterally implanted lead used has to be long enough (minimum suggestions: 69 cm for His and left bundle branch area pacing, 85 cm for CS pacing, 75 cm for right ventricular pacing, 65 cm for right atrial pacing) to allow tunnelling back to the original implant side The tunnelling tool must have a diameter sufficient to permit an IS-1, IS-4, or DF-4 connector pin to pass through (DF-1 leads are more problematic due to the yoke). Lead implantation is performed conventionally from the contralateral side. Then, a blunt-tipped tunnelling device (e.g. equipment used for ventriculoperitoneal shunts or the tunnelling tools used for either subcutaneous or extravascular ICD implantation) is gently pushed subcutaneously, suprasternal from the contralateral site to the initial device pocket. The lead can then be passed or pulled through to the initial device side (*Figure 8*).

Contralateral tunnelling is easiest with thin leads, e.g. for conduction system (Medtronic SelectSecure 4.1F) or CS pacing (5 Fr) but can also safely be performed in thin patients with 7 Fr pacing or 8–9 Fr ICD leads.

### Lead extraction to create venous access or manage redundant leads

Lead extraction can be used to facilitate vascular access where previously implanted leads and venous stenoses do not readily permit the addition of new leads. Lead extraction is a potential option during an upgrade procedure to remove leads that would otherwise be abandoned. This reduces the total number of leads left in the venous system and in the heart. In the scenario of challenging vascular access on the ipsilateral side, lead extraction to gain vascular access has a recommendation in the 2017 HRS expert consensus statement.^134^ Alternatives to lead extraction in this scenario include implanting additional leads on the contralateral side with subcutaneous tunnelling to the ipsilateral device or the implantation of a new system on the contralateral side with abandonment of ipsilateral leads. These non-extraction strategies may be suboptimal when considering lifetime lead management, particularly in younger patients with a long life expectancy.

In the scenario where extraction is not specifically required to facilitate vascular access, extraction is a potential option to remove redundant leads. Equally, these leads can be abandoned. Both strategies are considered valid with a recommendation in the 2017 HRS expert consensus statement.^134,173^ In these scenarios, the management strategy should be individualized through a shared decision-making process. There is no difference in published 10-year survival probability between both lead management approaches. However, it has been shown that lead abandonment may result in more complicated future lead extraction procedures if eventually required.^174^ This could impact ∼10% of patients with abandoned leads.^175^ Nonetheless, lead extraction in this setting is associated with an increased risk of acute procedural complications, including mortality.^36^ These recommendations stem from observational datasets, and as such determining the optimal strategy is currently uncertain.

There are two significant considerations with TLE during upgrade procedures:

1. The risk of collateral lead damage to other leads and
2. the potentially higher risk of peri-procedural complications.

In a retrospective single-centre study, out of a total cohort of 425 patients, 88 patients underwent lead extraction procedures for non-infectious indications.^176^ Of these 88 patients, 36 had venous occlusions and 49 had non-occluded veins (3 with unknown status). Patients with venous occlusion had higher odds for minor peri-procedural complications (12/36 patients compared with 2/49). However, major complications, including peri-procedural death, were not significantly higher in patients with venous occlusion (2/36 and 0/49 for major complication, *P* = 0.17 and 2/36 and 1/49 for peri-procedural mortality, *P* = 0.38). The risk of collateral lead damage ranges between 2.7% and 3.8% in a general lead extraction population.^177,178^ However, in the specific situation of TLE for recanalization, in cases of venous occlusion, the risk of collateral lead damage may be higher. In a retrospective single-centre study with 45 patients and 107 leads, 77 leads were targeted for extraction to re-canalize a venous occlusion with 30 bystander leads. Collateral lead damage was reported in 2 out of 30 leads.^179^

In contrast, results from the Multicenter Swiss Lead Extraction Registry have shown that TLE procedures during device upgrade can be performed effectively and do not add a disproportionate risk to the upgrade procedure.^100,167^ In the BUDAPEST-CRT trial, which showed significant benefit from upgrade, 15% of the CRT-D procedures and 11% of the ICD procedures required extraction.

## Alternative (non-superior access) delivery approaches

### Surgical/epicardial approach

In cases of complex venous anatomy, missing venous access, prosthetic tricuspid valve, or recurring infections, surgical implantation of epicardial leads or even complete epicardial CIED systems is an option.^180,181^ In most cases, epicardial lead placement may and should be achieved by minimally invasive approaches avoiding full sternotomy.^150,181^ Surgical approaches to upgrade can also be assessed and adopted concomitantly at the time of planned cardiac surgery.

Nowadays, implantation of complete epicardial systems remains a rare necessity due to the emergence of techniques detailed above. However, in the context of CIED upgrades, the minimal-invasive implantation of LV epicardial leads is a potential option that may be encountered in cases of failed vascular access or failed transvenous implantation of a CS lead. It has been shown that the use of an epicardial left ventricular lead results in equal improvement of LVEF and NYHA class in CRT.^151,152^ Furthermore, the electrical performance of epicardial leads has been shown to be non-inferior to transvenous leads in a series of more than 1000 leads over a 5-year period.^151^ It should be noted though that it can be a challenge when utilizing a minimally invasive approach to ensure placement of the epicardial lead in a postero-lateral position when aiming to deliver CRT. Optimization of approaches may be able to improve outcomes.^138^

### Leadless, endocardial, and other non-transvenous approaches

Femoral/abdominal, surgical/epicardial approaches are associated with additional ongoing morbidity as well as surgical risk; however, leadless pacemakers offer a lower-risk alternative approach for patients who have superior access issues and/or increased risk of infection, lead fracture, tricuspid valve or pocket complications.

Until recently, only single-chamber ventricular pacing systems were available to provide ventricular pacing support (Micra®, Medtronic, USA).^139,140^ Recent advancements have expanded leadless pacemaker technology to patients who would benefit from AV synchrony (AV Micra®, Medtronic, USA) and dual-chamber AVEIR® (Abbott, USA),^140^ and also for those who need atrial pacing (e.g. sinus node dysfunction) (atrial or dual-chamber AVEIR®, Abbott). Implant-to-implant communication between an atrial and a ventricular leadless pacemaker has been shown to be reliable. Dual-chamber leadless pacing provides AV synchrony for patients with heart block and sinus rhythm, rather than just best serving patients with persistent AF.^142^ Still, the long-term experience with these systems is yet to be demonstrated.

For patients with pacemakers *in situ* (transvenous, epicardial, or leadless) who develop a need for tachyarrhythmia support but have vascular access problems or an ongoing desire to avoid placing transvenous devices, extravascular ICDs (S-ICD and EV-ICD) can provide an alternative upgrade option. This scenario can lead to the addition of a separate device to the initial pacing device.

The subcutaneous ICD (S-ICD, Boston Scientific, USA)^143^ has been shown to be non-inferior to the transvenous ICD with respect to device-related complications and inappropriate shocks in ICD patients without an indication for pacing.^144^ Utilization of the S-ICD (Boston Scientific, USA) in pacing-dependent patients treated with a pacemaker has been explored and has been shown to be an effective strategy.^145^ However, patients require two separate systems, which can pose device interaction issues. Although extremely unlikely, the key priority is to ensure that the S-ICD does not misinterpret pacing spikes or oversense T waves as a tachyarrhythmia and thus may require careful programming of both devices.

Combination of an S-ICD (Boston Scientific, USA) with a leadless pacemaker may also facilitate the ability to deliver commanded anti-tachycardia pacing (ATP) using unidirectional wireless communication. The Modular ATP® system (Boston Scientific, USA) has been shown to have 61.3% successful VT termination.^146^ This technology is currently not yet approved by regulatory agencies. The S-ICD has also been shown to be feasible in combination with an epicardial CRT-D.^147^

An alternative substernal extravascular ICD (Aurora EV-ICD™, Medtronic, USA) is now available. There is minimal experience using this strategy for upgrading from a pacemaker at present.^148^ The EV-ICD (Aurora EV-ICD™, Medtronic, USA) can provide pause-prevention pacing, ATP, and defibrillation energy similar to that of transvenous ICDs.^149^ ATP success with this device has been estimated at 77%.^153,154,182^ Pacing capture thresholds are relatively high (4.9 ± 2.0 V at implant, 5.5 ± 2.0 V at 6 months; capture at ≥10 volts in 49 (16.4%) patients), and patient discomfort during stimulation has limited the use of pacing in some patients. It is worth noting though that the device is not designed primarily to provide frequent anti-bradycardia pacing.

Although the experience is limited, case reports exist where a patient develops a pacing indication leading to upgrade of an EV-ICD by adding in a dual-chamber pacemaker with good effect.^148^

For patients who require CRT and have vascular access problems, there is ongoing investigation with wireless technology that can provide LV endocardial stimulation synchronized with concomitant RV pacing using ultrasound-activated stimulation WiSE-CRT® (EBR Systems, USA) (Leadless-CRT).^155^ Initial experience with this technology shows favourable clinical responses in HF symptoms and LV reverse remodelling.^156^ Totally leadless CRT was delivered successfully with a combination of Micra® (Medtronic, USA) and WiSE-CRT® (EBR Systems, USA) in eight patients, and this application may potentially broaden the field of leadless cardiac pacing.^183^

### Femoral approach for lead placement

**Figure 9:**
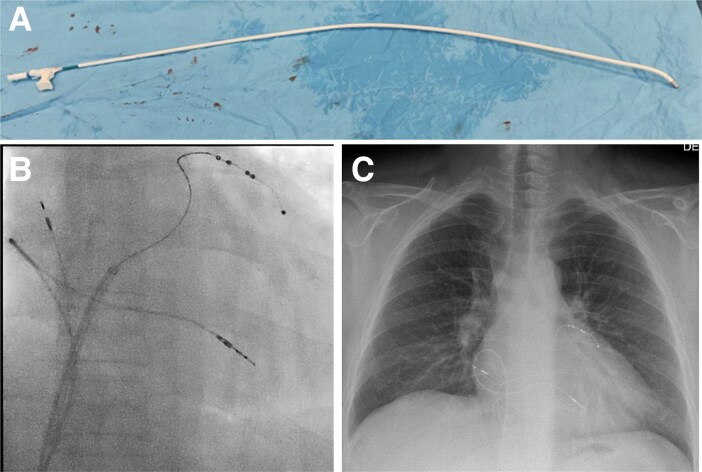
Implantation of CRT pacemaker via femoral access. Due to the coronary sinus (CS) delivery sheaths being too short, an SL-0 trans-septal sheath was customized by cutting off the hub and inserting a foreshortened Medtronic Attain CS sheath with a slittable hub (*A*). The CS was engaged using a deflectable diagnostic electrophysiology catheter, which allowed placement of the customized delivery sheath in the CS. A Medtronic Attain Stability (88 cm) active-fixation lead (Medtronic, USA) was positioned in an antero-lateral CS tributary, after having placed a Medtronic 3830 (74 cm) lead in the right atrium and a 5076 (85 cm) lead in the right ventricle (*B*). Final lead positions are shown in *C*.

**Figure 10:**
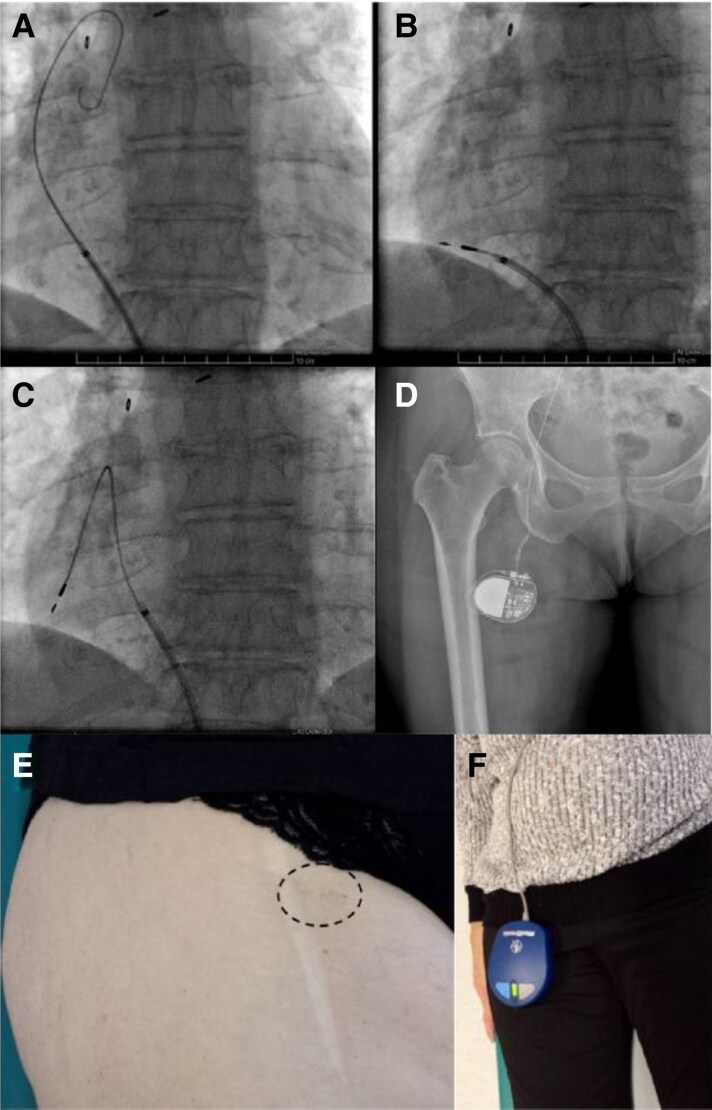
Femoral AAIR pacemaker implantation in a patient with occlusion of superior venous access after radiotherapy. Femoral venous access was obtained below the inguinal ligament, with positioning of a Medtronic 3830-69 lead via a C-304 deflectable sheath and fixated in the right atrial appendage (*A–C*). The pacemaker pocket was positioned in the inner thigh and fixated to the muscular plane (*D*). The incision healed well (dotted circle, *E*). Pacemaker interrogation with positioning of the telemetry head on the thigh (*E*). Figure reproduced with permission from Voirol *et al*.^184^

The ilio-femoral approach for pacemaker implantation has been described since the 1980s^160^ and is an option in case of upper venous access issues. The largest series to date included just 27 patients with a mean follow-up of 3 years.^158^ Venous access has been described either above or below the inguinal ligament. The former avoids lead crush and damage due to flexion of the hip, but exposes the patient to intestinal lesions and peritoneal bleedings, whereas the latter is simpler and probably safer as it is routinely performed using ultrasonography. It is necessary to use long leads (e.g. the Medtronic 5076 85 or 110 cm leads, or 3830 69 or 74 cm leads, or Abbott Tendril 2088TC 100 cm lead). Coronary sinus leads for CRT may also be implanted, but require customizing the delivery sheath (see *Figure 9*). The generator pocket can be placed subcutaneously in the inferior abdominal wall or in the inner thigh (which is simpler and is usually well tolerated—see *Figure 10*), with fixation of the generator to the muscular plane to avoid migration.

The most frequently reported complication is atrial lead dislodgement, which is described in up to 20% of patients.^158^ In order to reduce tensions on the lead, adequate slack with a loop in the atrium may be formed.^184^

Deep vein thrombosis/occlusion has not been described to date. With the advent of leadless pacing, this approach is likely now to be rarely used, other than potentially for CRT delivery.

**Table of advice:** Vascular access consideration

Vascular access considerations	Strength
**Advised to do**	
Superior (cephalic, subclavian, and axillary) vascular access strategies are advised over non-superior access strategies (i.e. femoral transvenous implantation)	>90% of writing group agree
Good quality contrast venography from the ipsilateral arm is advised prior to skin incision to assess the patency of the vein to help guide the procedure	>90% of writing group agree
Venous puncture is advised in an extra-thoracic location rather than using more medial punctures to avoid risk of pneumothorax and potential future lead failures^127^	High quality, large observational studies
**May be appropriate to do**	
When possible, venography may be useful to be performed in advance of the planned upgrade date to help risk-stratify and plan an upgrade approach	>90% of writing group agree
It may be appropriate that centres undertaking device upgrades have access to operators with skills to perform multiple techniques to cross stenoses, including venoplasty	>90% of writing group agree
Venoplasty, where possible, may be appropriate to facilitate venous access and enable device upgrade in case of venous obstruction	>90% of writing group agree
Lead extraction may be appropriate to obtain venous access and enable device upgrade in case of venous obstruction	>90% of writing group agree
Where superior venous access is challenging, alternative approaches may be appropriate, including S-ICD/EV-ICD, leadless pacing, and epicardial/surgical systems	>90% of writing group agree
Contralateral lead implantation and tunnelling may be an appropriate approach to add leads	>90% of writing group agree
**Advised not to do**	
Placement of more than 5 leads in the SVC and 4 from a single subclavian vein is not advised	>90% of writing group agree
Ultrasound is not advised as a first choice/optimal technique for assessment of venous patency to plan procedural vasculature access	>90% of writing group agree
**Areas of uncertainty**	
It is uncertain in which patients extraction of abandoned leads should be utilized when extraction is not required to facilitate vascular access	>90% of writing group agree

## Surgical considerations

Although the benefits of upgrading may exceed the risks, optimal surgical approaches can reduce the associated risk rate.

Complications include bleeding, haematoma formation (which can prompt infection), lead damage, infection, and scarring, and these may be mitigated with optimized surgical technique and tools.

### Consideration 1: timing of skin incision and pocket opening in relation to vascular access for cardiac implantable electronic device lead placement

**Table 7:** Summary of evidence for interventions aimed at infection prevention

Intervention	Study design	*n*	Main results
Pre-procedural antibiotics^223^	RCT	649	Trial stopped early by the safety committee due to a significant reduction in the risk of CIED-related infection (0.64% in the cefazolin group and 3.28% in the placebo group; *P* = 0.016)
Chlorhexidine vs. iodine-povidone for skin preparation^195^	RCT	849	Chlorhexidine resulted in a lower risk of surgical site infection, particularly superficial (4.2% vs. 8.6%, *P* = 0.008) and deep incisional infections (1% vs. 3%, *P* = 0.05) and deep incision infections in patients undergoing clean-contaminated surgeries Although not directly tested in CIED-related procedures, chlorhexidine may be the preferred choice in CIED upgrades
Antibacterial envelope^203^	RCT	6983	Compared to standard of care, the use of an antibacterial envelope resulted in a significant reduction in CIED-related infections (hazard ratio, 0.60; 95% CI, 0.36–0.98; *P* = 0.04)
Adherent iodine drape^196^	OBS	14 225	Use of an adherent skin drape was associated with a lower risk of CIED-related infection (OR: 0.32; 95% CI 0.154–0.665; *P* = 0.002) Possible differences in the baseline characteristics between groups (i.e. drape vs. no drape) are not ascertained
Chlorhexidine vs. normal saline lavage^198^	OBS	1504	Chlorhexidine lavage was associated with a significant reduction in CIED-related infection (hazard ratio 0.138; 95% CI 0.04–0.45; *P* = 0.001)

A number of approaches exist in clinical practice. In scenarios where a generator replacement (ERI) is not required and the proposed procedure is for new lead placement, some operators will aim to gain vascular access and puncture through the skin rather than making a skin incision. If vascular access is achieved, an appropriate incision is then made either pre- or post-lead deployment. If unsuccessful in gaining vascular access, the case can be abandoned at this juncture without having made an incision. The risk of infection from this approach is unknown as whilst no incision has been made, skin flora may still be introduced to the tissues and bloodstream via the puncture needle.

An alternative approach involves performing the skin incision upfront and to free the existing generator from its pocket prior to attempting vascular access. This approach means that once the additional lead is implanted, there are no additional surgical steps of the procedure to complete, and then an efficient process can follow to secure the lead and connect to a generator and place in a pre-formed pocket. This approach allows any inadvertent pocket bleeding to manifest early and be addressed well in advance of wound closure and potentially reduces the risk of lead displacement during subsequent pocket manipulation and intervention. Furthermore, removing the generator from the pocket allows better visualization of the puncture site using fluoroscopy. However, the generator and leads are unnecessarily disturbed if vascular access/upgrade is not successful in this scenario, and furthermore, exposed to potential contamination for a longer period.

In cases where a generator replacement will be carried out (ERI), one might consider to first make the skin incision, and then free the existing generator and modify the pocket as required before seeking vascular access. Alternatively, one might think to obtain vascular access and lead placement percutaneously prior to skin incision as this may reduce the length of time the generator pocket is open. Data determining whether one strategy is more favourable than another is lacking.

### Consideration 2: location of incision

Whilst for *de novo* implants there are three incision types (deltopectoral, horizontal, and oblique), the incision for a device upgrade (which involves addition of a new lead on the ipsilateral side) should be optimized for venous access. The location of the initial incision and scar and additionally the location of the generator should be a secondary consideration. This is particularly relevant if the initial implant utilized a deltopectoral incision and the cephalic vein and/or if the generator has since displaced caudally. It is often worthwhile to screen the location of the incision using an instrument held over the skin to ensure the location of the incision is in fact optimal for gaining vascular access. Furthermore, if lead extraction is being considered, the incision may need to be adjusted to allow coaxial alignment of lead extraction tools.

### Consideration 3: surgical techniques; blunt dissection vs. diathermy vs. low-thermal-injury dissection device

Traditional diathermy or blunt dissection is associated with greater risks of complications due to excessive thermal injury (to tissues and/or leads) or less precise dissection, particularly close to the existing leads.

While the use of diathermy has been maintained in some centres, many centres have migrated to the use of low-temperature electrocautery devices, such as pulse electron avalanche knife (PEAK) PlasmaBlade™ (Medtronic, USA) or PhotonBlade (Invuity, USA). These single-use, low-temperature surgical instruments are powered by a specialized radiofrequency electrosurgical generator (AEX^TM^, Medtronic, USA) or a standard electrocautery generator, respectively. The instrument uses brief (∼40 μs) pulses to induce electrical plasma along the edge of a 12.5-μm-thin insulated electrode, allowing it to operate at low temperatures in the range 40–170°C. With standard cautery (diathermy), the high temperatures are in the range of 200–350°C.^185,186^

Kypta *et al.* retrospectively compared two groups: 509 patients with scissors and conventional diathermy with 102 patients who had PlasmaBlade™ (Medtronic, USA). They found shorter procedure duration and less major complications, including infection and lead damage (2.4% vs. 6.9%), leading also to demonstration of cost reduction.^186,187^ In a sub-study of WRAP-IT, PlasmaBlade™ (Medtronic, USA) was associated with a 23% reduction in procedure or lead-related adverse events during 3 years of follow-up, and when controlling for complexity of the case the risk reduction improved further with a 32% lower risk of complications (HR 0.68; 95% CI 0.52–0.89, *P* = 0.004).^187^ While no high-quality results from RCTs exist on this topic, these data from observational studies and post-hoc analysis of data from an RCT suggest PlasmaBlade™ (Medtronic, USA) may be associated with fewer complications and might support its use over conventional diathermy. PhotonBlade (Invuity, USA) has been compared in bench experiments with PlasmaBlade™ (Medtronic, USA) to show less lead damage reported.^188^ In a small retrospective observational study, similar outcomes were observed between the two technologies.^189^

### Consideration 4: capsulectomy

Even in the absence of signs of clinical infections, cultures taken at the time of generator change demonstrate a significant incidence of bacterial colonization.^190^ In addition, the biofilm-prone fibrous capsule inhibits the body's normal defence mechanisms and antibiotic penetration. Theoretically, ‘capsulectomy’ might therefore mitigate these issues. However, if capsulectomy is performed, the risk from resultant pocket bleeding and subsequent haematoma risk is significantly elevated. In a 659-patient observational study, pocket haematoma was associated with a ∼7-fold increased risk of pocket infection (hazard ratio: 7.7; 95% CI: 2.9–20.5; *P* < 0.0001).^106,191^ It is therefore advised not to perform capsulectomy as routine practice.^192^ Partial capsulectomy may have a role in the presence of a highly fibrotic capsule causing suboptimal device positioning, but limited data exist addressing this.

### Consideration 5: management of abandoned leads

Where the upfront decision is to abandon a pacing lead rather than proceed with lead extraction (in the scenario where lead extraction is not required for vascular access), proper management of the abandoned lead within the pocket is essential. This is to reduce infection rates (abandoned leads have previously been shown to have higher infection rates than when extraction is performed),^173,193,194^ to prevent lead migration, lead erosion, interference with other components of the CIED system (leading to noise and inappropriate device behaviours), and also giving consideration to later extraction of the lead if this becomes necessary.

The abandoned lead should be coiled and placed deep within the device pocket to avoid interacting with other CIED components. Best practice often involves looping the lead within the bottom of the pocket. Some operators choose to anchor it in place with a suture to reduce its risk of migration.

Two approaches are commonly utilized when abandoning a lead. The first approach is the Cap and Bury method. Here, the lead end is covered using a specialized cap, which helps to prevent exposure of the conductive elements and reduces the risk of fluid or debris entering the lead, aiming to reduce infection risk. It is essential to ensure the cap is secured with at least one suture, ideally fixated to the muscular plane to avoid migration to the surface. This approach keeps the inner conductor clean (minimizing debris accumulation) and therefore allows the leads to be extracted readily if needed in the future. There may be an advantage of this approach if future extraction is required due to longer available lead length.

The second approach is to use the insulation pull-back approach. In this approach, the lead is cut and the insulation of the lead is pulled over the proximal end aiming to cover the exposed conductor. For greater protection and to avoid entry of fluid into the lead lumen (which may compromise future use of locking stylets for extraction), the lead end is knuckled (bent back upon itself) and firmly tied using non-resorbable suture material before being fixated to the muscular plane. This approach reduces the volume of the pocket; it is important though to try to leave sufficient lead length for potential future extraction should this need arise.^98,134^

### Consideration 6: infection prevention and antibiotic envelopes (*Table 7*)

Pre-procedural antibiotic use particularly ensuring cover against *Staphylococcus aureus* has been shown to reduce infection rates in all device procedures and has now become an absolute requirement.^38,98^ Chlorhexidine-alcohol skin preparation has been shown to reduce surgical site infections over iodine-based products in non-CIED surgery and should therefore be a preferred skin cleansing approach for device upgrade or downgrade procedures.^195^

Adherent drapes (with or without iodine coating) have been evaluated in 14 225 patients in an observational study and were observed to be associated with lower infection rates compared to no drapes (odds ratio 0.32; 95% CI 0.154–0.665; *P* = 0.002).^196^ However, there was no separate reporting of results for the subgroup of patients with upgrades, or of efficacy of iodinated vs. non-iodinated drapes. The results of an RCT assessing the effectiveness of using a barrier-adhesive-draping on reducing the end-of-procedure pocket swab culture are pending.^197^ Pocket lavage has been associated with reduced infection rates with a large observational study showing that chlorhexidine has even been associated with lower infection rates compared to normal saline lavage.^197,198^ Short post-operative antibiotic regimes have failed to demonstrate incremental benefit, whereas longer regimes showed possible potential incremental benefit, but only limited supporting data are available.^199,200^ An additional dose of intraprocedural antibiotics may be appropriate if the duration from the time of antibiotic prophylaxis administration plus procedure time is >240 min or if the duration of the procedure exceeds 2 half-lives of the drug.^201,202^

### Antibiotic envelopes

The addition of antibiotic envelopes to the current pre-operative intravenous antibiotic prophylaxis regimen can be useful in lowering the rate of pocket infection. There are currently two commercially available envelopes—*TYRX^TM^* (Medtronic, USA) and the *CanGaroo®* (Elutia Inc., USA).

The *TYRX^TM^* (Medtronic, USA) antibiotic envelope is an antibiotic-impregnated mesh sleeve that releases rifampicin and minocycline over a minimum of 7 days and is fully absorbed within 9 weeks. The Worldwide Randomized Antibiotic Envelope Infection Prevention trial (WRAP-IT) is the only randomized clinical trial of antibiotic envelopes to date.^203^ The study demonstrated a 40% reduction in major infections (HR 0.60, 95% CI 0.36–0.98), (mainly pocket infections) within 12 months. The rate of infection was, however, low (1.2% vs. 0.7% events), thus the number needed to treat is high at 200 patients, although this number may be lower in patients at higher risk, such as those undergoing device upgrade procedures. The benefit of *TYRX^TM^* (Medtronic, USA) was sustained beyond the first 12 months (mean 21 ± 8.3 months) with a reduction in CIED infections of 1.9% vs. 1.3% (HR 0.64 95% CI 0.41–0.99).^109^ These results have been emphasized in a recent meta-analysis and other retrospective and observational studies, which all present a significant risk reduction in CIED reoperations with *TYRX^TM^* (Medtronic, USA).^204,205^

The 1010 patient ENVELOPE RCT demonstrated no incremental benefit of antibiotic pocket irrigation and post-operative oral antibiotics beyond prophylactic measures of chlorhexidine skin preparation, pre-operative intravenous antibiotics, and an antibiotic envelope in reducing CIED infection in high-risk patients (mean PADIT score 7.4).^206^

Clinical risk prediction tools such as the PADIT and BLISTER scores have been developed to guide infection prevention strategies. Both incorporate key patient-level risk factors, such as renal dysfunction, prior procedures, and device type, but differ in the inclusion of variables, like immunosuppression (BLISTER) and timing of prior procedures (PADIT).^199,207,208^ However, neither score accounts for procedure duration nor complexity, such as difficulty with vascular access. These factors may also influence infection risk and inform the decision to use an antibiotic envelope and indeed in a European survey of 301 physicians, only 49% used a risk score to support their decision whether to use an antibiotic envelope or not.^209,210^

The utilization of *TYRX^TM^* (Medtronic, USA) comes with an incremental cost and is currently not suggested in all CIED reoperations. However, it seems reasonable to consider the use of the antibiotic envelope in:

1. Patients with the highest risk of CIED infection over time. Several studies have associated young age with a higher risk of infections, probably in part due to multiple reoperations and a lower competing risk of dying than in the elderly population.
2. Patients who are at the highest risk for adverse outcomes from CIED infection, such as those with CRT in whom re-implantation of an LV lead may be very challenging or technically unfeasible following extraction, individuals with prosthetic valves who are vulnerable to prosthetic valve endocarditis secondary to systemic infection, or patients in whom lead extraction carries significant morbidity.^32–35,134^
3. Patients in whom the incremental cost-effectiveness ratio supports the usage of antibiotic envelopes based on the gained quality-adjusted life years compared to the standard of care.^211^
4. Patients undergoing CRT-reinterventions particularly with previous interventions in the same pocket.^43,204^

In the cost-effective analysis of the WRAP-IT study, high cost-effectiveness was demonstrated in immunocompromised patients, those with high-power devices, two or more previous procedures, as well as in revision or upgrade of low-power devices.^212^ In an additional analysis of WRAP-IT, related to the context of European countries, the absorbable antibacterial envelope was associated with cost-effectiveness ratios below European benchmarks in selected patients at increased risk of infection, suggesting economic value for the European healthcare systems when used in these patients.^213^

The *CanGaroo®* (Elutia Inc., USA) is a biologic envelope that is hydrated in a solution of gentamicin before implantation, and ensures an early peak concentration that prevails for up to a week.^214^ In a pre-clinical setting, it has demonstrated promising findings in reducing bacterial burden.^215^ In the 1025 patient, single-arm SECURE study (NCT 02530970; results available on clinicaltrials.gov) 1.2% had a major pocket infection over an average follow-up period of 235 days. These data are limited though due to the lack of a control group.

Compared to this extracellular matrix envelope, pockets with TYRX™ showed less inflammation, more rapid provisional matrix formation, faster absorption, and thinner capsules.^216^ The extracellular matrix envelope does not seem to reduce the incidence of device skin erosion.^217^ Consequently, there is not enough information available to advise using the *CanGaroo®* (Elutia Inc., USA) extracellular matrix envelope currently.

Taurolidine has been shown to destroy pathogens, impede surface adhesion, neutralize endotoxin and exotoxins, and promote wound healing.^218–221^ It is a derivative of the non-essential amino acid taurine. Taurolidine is unstable in aqueous solution and breaks down into derivatives, which are responsible for its biological activity.^220^ N-methylol groups released during the process chemically react with the amino and hydroxyl groups of susceptible molecules in the cell wall of pathogens and certain toxins, denaturing the endotoxins and polysaccharide/lipopolysaccharide components in the cell wall of pathogens thus deactivating susceptible exotoxins.^222^

The use of taurolidine solution (*TauroPace^TM^* Tauropharm, Germany) as an intraoperative antimicrobial solution adjunct (AMSA) for combatting CIED infection was studied in an observational study.^222^ The incidence of infection using taurolidine solution was compared to historical controls using hydrogen peroxide (H_2_O_2_) (1205 CIED procedures). Taurolidine solution was observed to be associated with lower rate of acute device infection than H_2_O_2_ (0% vs. 1.1% respectively, *P* = 0.0075).^222^ However, these data were observational in nature, and randomized controlled clinical trials with long follow-up are warranted to confirm these results. Until then, it cannot be advised to use taurolidine for CIED upgrade procedures.

**Table of Advice:** Surgical considerations including infection risk reduction

Surgical considerations including infection risk reduction	Strength
**Advised to do**	
Utilization of an antibiotic envelope is advised in patients at higher risk of infection^109,203,205,212^	High quality, large observational studies
**May be appropriate to do**	
Utilization of low-temperature electrocautery techniques may be appropriate over regular diathermy and blunt dissection where available	>70% of writing group agree
The cap & bury approach may be an appropriate management approach for abandoned leads. The insulation pull-back approach may be an appropriate management approach for abandoned leads	>90% of writing group agree >70% of writing group agree
It may be appropriate to coil and secure abandoned leads within the pocket to reduce risk of interaction with other pacing components and erosion	>90% of writing group agree
**Advised not to do**	
Capsulectomy is not routinely advised due to risk of possible resultant haematoma	>70% of writing group agree
**Areas of uncertainty**	
It is unknown whether during an upgrade procedure (without a need for generator replacement), if there is a benefit to obtaining vascular access prior to skin incision and opening the pocket, or whether alternatively, making a skin incision and opening the pocket is a preferable first procedural step	>90% of writing group agree

### Patient perspective

When considering a CIED upgrade from the patient's perspective, several key aspects must be addressed to ensure clarity and manage expectations.

Patients may feel overwhelmed and anxious when informed that their current device is no longer sufficient for their clinical needs and that an upgrade might be necessary. This anxiety can often be offset by explaining technological advances have occurred, facilitating these recommendations, which possibly may not have been available at the time of initial device implant.

It is crucial to discuss the potential for vascular access issues with patients upfront, along with alternate solutions and their advantages and limitations. When preparing for the procedure, it is important to have an open dialogue about the timing of venography. Conducting venography in advance of the procedure date can offer clarity by helping delineate with greater certainty expectations and solutions, which can then be discussed with the patient. This proactive approach helps to manage expectations and ensures patients feel informed about their options. Although this upfront approach enables better planning for the medical team and clarity for the patient, logistically, it is not always an available option with many patients having their venogram only on the day of the procedure.

It is crucial to discuss with each patient available options for delivery of additional leads. It is important to consider a patient's occupation and physical activities as contralateral implantation may impact upon their dominant side, which could affect their physical activities or raise cosmetic concerns due to multiple chest wall scars.

Lead extraction (where considered as an option) should be explored in detail with the patient. Patients need to be aware of and appraise the risks and benefits associated with lead removal as opposed to lead abandonment or alternative approaches. Individual patients may have different perspectives on acceptable risk.

## Considerations for individualized risk–benefit analysis

As with any clinical decision, clinicians must analyse the risk–benefit profile of the patient with respect to any proposed device upgrade or downgrade.^13,59,111,134^

**Figure 11:**
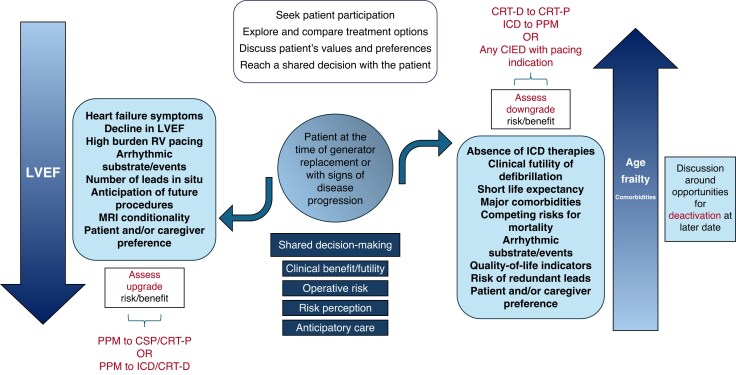
Suggests factors to evaluate in conjunction with a patient at the time of a planned generator replacement or if the patient shows signs of disease progression.

The risks will vary depending on multiple factors (patient-, procedural-, and system-related); these must be carefully calibrated to ensure that risks are minimized and decisions are made correctly and appropriately. These are summarized in *Table 8* and *Figure 11*.

**Table 8:** Outlines the various factors to consider when evaluating the risks and benefits of a procedure

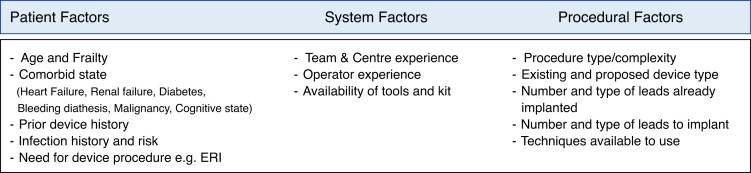

A generator change or end of battery life offers a key opportunity for clinicians to re-evaluate the suitability of a device and assess whether it is still aligned with a patient's current clinical requirements and prognosis as well as their individual preferences.^13,111,134^

When a patient's medical needs have changed and an up- or downgrade of their device is suggested, it is important to acknowledge that the patient's personal circumstances, preferences, expectations, risk perception, etc. may have changed too. These must be assessed again as part of the shared decision-making process.^224–229^

A change of device type might be prompted by a deterioration in the patient's cardiac condition. This information alone may be overwhelming for a patient, which might preclude immediate device upgrade or downgrade discussions. Repeat consultations may therefore be required perhaps with a family member or other advocate present to progress such discussions. At all clinic appointments, signposting possible device upgrades or downgrades as a potential future management strategy may better prepare patients for such scenarios. This can help manage patient expectations.

### Suggested framework

1. **Seek the patient's participation.** Explain why the implanted device no longer meets the needs of the patient. Encourage the patient to play an active role in the decision to keep, up- or down-grade the device. Suggest inviting caregivers to participate in the process.
2. **Help the patient explore and compare treatment options.** Provide an overview of all treatment options, including refusal, in a way that can be understood by everyone. Discuss the benefits and risks of each option. Provide patient educational materials and, if possible, decision-making aids to support the consultation. Give the patient enough time to consider the options.
3. **Assess the patient's values and preferences.** It is important to acknowledge that as their disease progressed or improved, a patient's personal circumstances, preferences, expectations, risk perception, treatment goals, etc. may have changed as well.
4. **Reach a decision together with the patient.**

#### Role of the multi-disciplinary team and ethical framework

Shared decision-making should be at the cornerstone of this process throughout.^13,224–229^

In the cohort of CIED downgrade, there will be some patients unable to input into the decision-making process (due to either temporary or permanent loss of mental capacity, for example in patients with dementia). In these circumstances, these discussions should be held in conjunction with those healthcare professionals who are most familiar with the patient's care and in the absence of any advance directives, also include any nominated persons (family, other next of kin, or legal representative if available) who will be able to advocate for the patient's best interests. The process of determining what is in the best interests of the patient must consider the patient's previously expressed wishes, beliefs, and values and should seek views from those who knew the patient best, to understand their priorities, where possible. If loss of capacity is temporary, the decision should be delayed, where possible, until capacity has recovered. Any best interests process should also be carried out in accordance with national guidance.

Situations where referral to a court of law or an Ethics committee are outlined in the ‘*EHRA consensus statement of management of CIED in patients nearing end of life or requesting withdrawal of therapy*’, and may be equally applicable in circumstances where a downgrade of a device is considered.^227^

In many countries, withdrawal of pacing therapy (for bradycardia indications) in pacemaker-dependent patients is contrary to the law; however, even where this is permissible from a legal standpoint, it is uncommon in practice, and the impact of continued pacemaker therapy in prolonging an inevitable dying process is unknown.^227^

The multi-disciplinary team should re-evaluate the indication for device therapy and any specific requirements. Repeat assessment of a patient's heart rhythm, need for CRT, or reliance on pacing should be assessed. Complete assessment may even require a short period of device reprogramming to a passive or backup only mode to enable information gathering i.e. whether a patient deteriorates rapidly without delivery of CRT therapy when considering device downgrade or discontinuation of therapy. The multi-disciplinary team should also evaluate whether programming changes (i.e. utilization of pacing avoidance algorithms) or medication-based strategies can be better utilized. It is unknown whether HF GDMT can be used as a sole alternative to device upgrade in the scenario of genuine PICM or when a strong indication for upgrade to CRT or ICD has developed.

#### Risk at generator change vs. any other time

The risk of infections or pocket-related complications of recurrent CIED replacements is incrementally elevated with each procedure.^43^ Early intervention prior to ERI of the original device may result in a patient having an additional device procedure in their lifetime with a potential subsequently elevated risk. However, cardiac resynchronization (if high burden of RV pacing/broad LBBB and LV impairment) or ICD (for secondary prevention of arrhythmia or if severe LV systolic dysfunction) upgrades should be performed in a timely manner to potentially avoid irreversible decline and to reduce morbidity and mortality.^13,111,134^ This should not be delayed until generator replacement or a later date to improve patient outcomes.^27,112^

In cases, however, where the indication is currently more nuanced (for example, patients with mid-range LVEF and only modest pacing burden), discretion should be used as this is currently an area of uncertainty and waiting till ERI might be more appropriate.

#### Specific situations

As already stated, risk–benefit evaluations for device upgrades and downgrades need to be individualized, with certain conditions warranting particular attention.^13,111,134,230^

In patients with a degree of frailty and/or comorbidity, assessment should be tailored to the individual, but should always include: frailty, functional status, care requirements, and patient support network (particularly relevant for recovery), comorbidity burden including polypharmacy, and cognitive status, including capacity to consent for a procedure.^230^

The multi-disciplinary team should seek to integrate these elements of the patient assessment, as well as the views of the patient and their advocates and those of other stakeholders, such as primary care providers and allied health professionals, to make an informed decision.

Certain conditions may need particular consideration:

**Advanced frailty:** By definition, a condition of impaired reserve, the physiological stress of device upgrade in someone with advanced frailty may represent an unacceptable risk to their already declining health, offsetting any potential benefit of device upgrade. Input from a geriatrician or other frailty practitioner can support the patient and the multi-disciplinary team in reaching a decision. Frailty scores may be a useful adjunct as an objective tool, particularly if serial measurements are available, but do not replace a complete clinical assessment.

Frailty may be associated with sarcopenia and/or low body-mass index (BMI), which may necessitate suturing of the device in place or intramuscular placement of the device. Sarcopenia may also cause greater than expected levels of pain related to the procedure and may increase the risk of device/lead erosion. The risk of erosion or infection can be elevated with the use of caps for abandoned leads. This strategy is, however, frequently preferred in this patient cohort in lieu of extraction. The careful placing of any abandoned leads therefore is advised.

Whilst in principle frail and older patients may be at higher risk of procedural complications, data from high-volume centres have shown that efficacy and complication rates are similar to those in younger, non-frail populations, provided additional care is taken. Therefore, referring such patients to experienced centres is crucial.^230^

**Long-term corticosteroid use** can be associated with skin atrophy, impaired wound healing, and elevate the risk of infections. Such patients are more susceptible to bacteraemia, longer hospitalization, and higher mortality rate. If procedures can be performed without concomitant steroid use, this is preferred.

**COPD or other severe pulmonary disease** may be associated with a higher risk of pneumothorax. Additionally, in those cases, when lead removal or extraction is planned, the risk of prolonged use of respiratory therapy is higher.

**Patients with diabetes** are at higher risk of device infection and prolonged wound healing; therefore, antibiotic envelope use may have potential benefit.^42^

**Patients with kidney disease** are also at higher risk of device infection and prolonged wound healing; therefore, antibiotic envelope use may have potential benefit.

When patients are treated with regular dialysis, venous access can be limited, and upgrades here can be associated with a 4-times higher risk of infection. Therefore, the use of leadless pacing and subcutaneous ICD is a potential option if clinically recommended.^13,111,134,145^

##### Device deactivation

**Figure 12:**
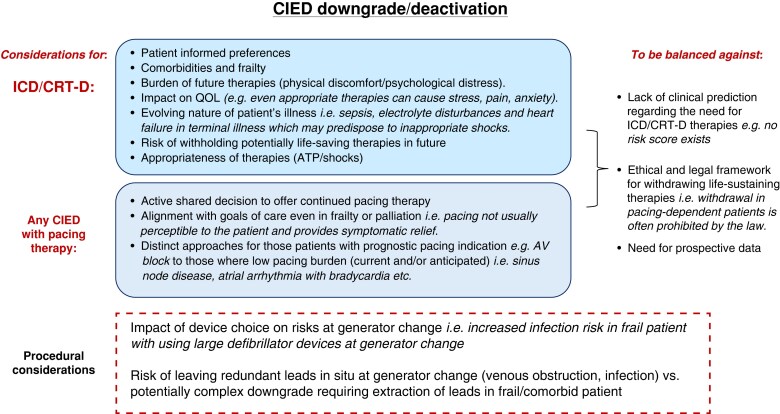
Framework for assessing CIED upgrade or downgrade decisions. ATP, anti-tachycardia pacing; QOL, quality of life; AV, atrioventricular; ICD, implantable cardioverter defibrillator; CRT, cardiac resynchronization therapy.

Evidence-based information on the topic of downgrade is limited. Observational data suggest reduced benefit of ICD therapy with increased age (likely due to competing risk of death from non-arrhythmic causes) and meta-analysis data suggest a non-minor procedural risk from ICD replacement (median rate of 4% for major complications).^40^ Ideally, any discussions around deactivation should be part of an advanced care planning process, which should involve caregivers, primary care, palliative care, and other multi-disciplinary team members, including geriatricians and specialist nurses (proposed framework outlined in *Figure 12*). It is important that the patient is given the opportunity to articulate their anxieties, which may include thoughts such as ‘*are you giving up on me?*’ and ‘*I was told the device is needed to keep me alive. If you switch it off, will I die?*’. Exploration of these and other concerns, in a sensitive and supportive way, using clear, unambiguous, jargon-free language, is central to the patient's experience at a time when they are likely to be feeling vulnerable. It should also be noted that the option of not replacing the ICD generator is worthwhile to broach at the time of original implant where appropriate and the decision to continue offering ICD therapy should be re-evaluated at follow-up. Outside of the end-of-life scenario, up to 15% of patients would consider electing not to proceed with ICD generator replacement when they are engaged in the decision-making process.^226^

**Table of Advice:** Individualized risk–benefit analysis

Individualized risk–benefit analysis	Strength
Advised to do	
Individual patient risk–benefit analysis before the upgrade/downgrade procedure is advised	>90% of writing group agree
In patients considered for CIED up- or downgrade, it is advised that risks and benefits of the procedure, the patient's preferences, and goals of care are explored in a shared decision-making process	>90% of writing group agree
It is advised that frailty, comorbidity burden, cognitive status as well as functional status, care requirements, and support network are assessed by the multi-disciplinary team with the involvement of caregivers to help guide device upgrade or downgrade decisions	>90% of writing group agree
If a device is deactivated/made non-functional, it is advised that the procedural risks of device removal and the risks associated with leaving the device *in situ* are assessed individually	>90% of writing group agree
**May be appropriate to do**	
It may be appropriate to prepare patients for potential decisions relating to their device early on in their device lifetime. This may help patients prepare for decisions relating to future upgrade, downgrade, or deactivation of their CIED	>90% of writing group agree
**Areas of uncertainty**	
It is unknown whether GDMT should be optimized first in the treatment of isolated pacing-induced cardiomyopathy (before proceeding to device upgrade) or whether earlier device upgrade should be performed potentially reducing the need for medication utilization	>90% of writing group agree

## Distinct case scenario discussions

**Figure 13:**
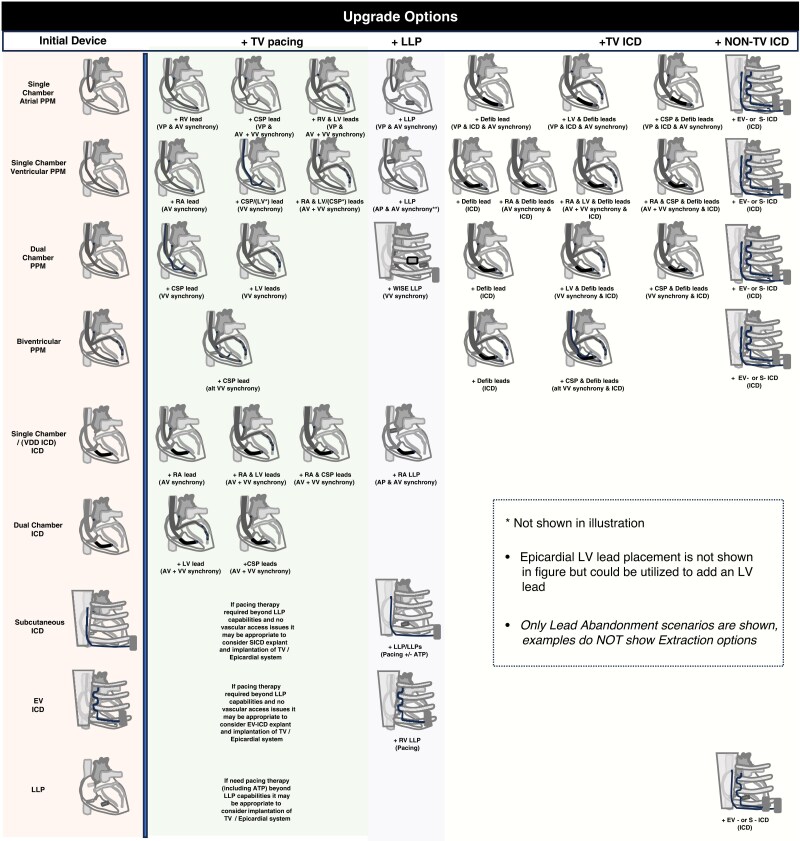
Overview of common upgrade scenarios.

This section explores commonly encountered scenarios and provides an overview of available upgrade options. *Figure 13* provides a graphical summary overview of common CIED upgrade scenarios.

*Table 9* outlines upgrade options and management strategies for patients with an already implanted transvenous pacemaker or ICD. In patients with a transvenous pacemaker, an upgrade may be appropriate if a patient develops a defibrillator indication. In both patients with transvenous pacemakers or ICDs, an upgrade may be appropriate to deliver additional pacing capabilities; for example an atrial lead for AV synchrony (if only a ventricular lead was initially implanted), or addition of a LV or CSP lead if a physiological pacing strategy is desired (due to either high burden of pacing or the development of an indication for resynchronization therapy for example LBBB and heart failure).

**Table 9:** Upgrade options and management strategies for patients with existing transvenous CIED

Existing CIED	Upgrade	Management strategies	Key points
Leaded TV CIED	Single-chamber to dual-chamber PPM (upgrade AAI or VVI PPM to dual-chamber PPM)	If VVI system add a transvenous RA lead and use the existing RV lead for a DDD pacemaker If VDD system abandon the RA sensing port and add a transvenous RA lead. This may impact MRI access in some centres If AAI system add a transvenous RV lead If single-chamber PPM, dual-chamber leadless pacemaker (Aveir DR system, Abbott, USA), or Aveir single-chamber device to co-exist with leaded CIED to provide dual-chamber system or AV Micra® (Medtronic, USA) can all be utilized if vascular access issues preclude implantation of new atrial or ventricular lead	If initial lead is a VDD lead,^231–233^ could consider lead extraction and replacement with a standard RV bipolar lead for example to achieve formal MRI-conditionality, but this would not be a common first-line approach in non-infected systems
	Upgrade from single or dual chamber PPM to CRT-P/CSP pacing strategy	Addition of either an LV lead to an RV pacing system or addition of a CSP lead to an RV pacing system Where a CSP lead is added, consideration can be given whether to incorporate the original RV lead within the new pacing approach or whether to abandon the original RV lead. If original system is VVI, it could incorporate CSP upgrade within a dual-chamber device (utilizing the atrial port) or a CRT-P device with a plugged atrial port	Upgrading to a physiological pacing strategy may be relevant in patients with pacing-induced cardiomyopathy or for those with broad LBBB If upgrade to CSP is performed, both the existing RV lead and CSP can be incorporated into the new device (typically CRT-P generator). Whilst a CRT-P costs a little more than a DR PPM, this cost is frequently not prohibitive. This may be useful rather than abandoning leads The decision to abandon leads is patient-specific and will take into account individual considerations, including the risk of extraction and the patient's vasculature In the case of pacing-induced cardiomyopathy resulting in severe LV impairment (no other cause for LV deterioration identified), the added net value of a defibrillator is not known and is an area of uncertainty. The LV function may well improve with improvement in cardiac activation from change in pacing approach. Retrospective cohort studies suggest >70% of patients with PICM will improve within 12 months to have an LVEF > 35%.^234^ In patients with EF <35% and with significant comorbidity and increased frailty, upgrade to a pacing only solution may be more appropriate than upgrade to CRT-D
	Upgrade from PPM/CRT-P to ICD or to CRT-D	Addition of a standard RV-ICD lead is the usual solution, but it will result in at least two leads traversing the tricuspid valve that could induce or worsen TR If a physiological pacing strategy is needed, addition of two leads will likely be required to a standard pacemaker: either a lead in the coronary sinus or at the conduction system plus the standard RV defibrillation lead In younger patients (particularly with pacing leads that have only been in for a short duration), it may be preferable to consider extraction of the RV pacing lead prior to ICD /CRT-D upgrade to reduce the number of leads in the heart as part of a lead management strategy If vascular access is challenging, an S-ICD can be used in conjunction with the existing pacing device, particularly in those who do not need ATP. This is a reasonable solution for primary prevention indications. There is evidence of efficacy and no increase in inappropriate shock.^235^ EV-ICD is a possible alternative if ATP is required, but long-term data remain limited.^149^ In both cases, the patients will need two devices, and MRI non-compatibility may be a concern. **ICD need only:** If there is patent venous access, implant RV bipolar ICD lead at alternative site within the ventricle. Subsequently, abandon or extract the existing RV lead. This will be the most common strategy. Implant a subcutaneous ICD as a co-implant if anticipated low ATP requirements^235^ Add an EV-ICD with ATP potential^149,236,237^ **ICD + CRT (BiV or CSP) need** Implant a standard CRT-D, either abandon or extract the RV pacing lead (an RV defibrillator lead and LV/CSP lead are added)	Considerations include the need for ATP, likelihood of lead-induced or worsening of TR and its impact, need for physiological pacing, and patient factors (age, comorbidity, etc.) A key consideration in this scenario is often whether to abandon the existing RV lead or extract it. When adding two leads, the RV pacing lead can be abandoned or extracted, but in younger patients, particularly where existing leads have only been in a short time, greater consideration should be given to extraction.^238^ Dedicated ICD leads that are able to provide CSP are being trialled and may be an option to reduce additional lead requirements contingent on ongoing trial results. Additionally, the role of repurposing existing ICD leads for deployment at the left bundle location is being investigated too.^239^ If an ICD is added without extracting the RV pacing lead, it may be worth considering whether the new ICD lead should be a dedicated bipolar lead rather than an integrated bipolar lead to avoid potential problems with sensing

Upgrade options include implantation of additional transvenous pacing or defibrillator leads if defibrillator capability is needed. Infrequently, leads outside of the vasculature can be utilized as part of the upgrade strategy [i.e. S-ICD (Boston, Scientific, USA) for defibrillator capability or leadless or surgical lead placement for pacing needs].

*Table 10* outlines upgrade options and management strategies for patients with a single-chamber leadless pacemaker. Upgrades may be appropriate to provide better atrioventricular synchrony and/or biventricular synchrony, which is not provided by the existing device. Upgrade may also be needed if the patient develops an ICD indication.

**Table 10:** Upgrade options and management strategies for patients with leadless CIED

Existing CIED	Upgrade to	Management strategies	Key points
Leadless pacemaker (LLP)	Leaded CIED (Pacemaker or Defibrillator)	Implant of transvenous ICD/pacemaker (single, dual, or CRT system) and deactivation of leadless pacemaker Implant of transvenous ICD/pacemaker (single, dual, or CRT system) and removal of the leadless pacemaker	Removing a leadless pacemaker when converting to a leaded system reduces the implanted material inside the heart—the benefit of this is, however, unknown. Removing LLPs poses challenges related to procedural complexity, risks, and costs. If the LLP is a Micra device (Medtronic, USA) and there is only one leadless device in the heart, it is often preferred in clinical practice to abandon the device (particularly, if longer dwell time and provided there is no evidence of LLP-related infection). Micra devices (Medtronic, USA) do not have a dedicated mechanism for retrieval, but can still be removed with a snare with high success rates.^240,241^ However, should the device be an Abbott NanoStim or Aveir (Abbott, USA) leadless pacemaker, these devices have a dedicated docking button for retrieval, and studies report a ∼90% retrieval success rate.^242,243^ Evidence of infection would necessitate explant/extraction of the device
	LLP + S-ICD or EV-ICD	Implant of S-ICD or EV-ICD system in addition to ongoing pacing provision from leadless device^145,244,245^	Consider the possible interactions between LLP and ICD. Broad RV paced QRS morphology can potentially lead to double counting of signals and therefore possible inappropriate shocks Intraoperative testing for interactions (ICD waveform screening during ventricular pacing and sensing with possible lead placement revision to ensure appropriate sensing) should be taken into account. For EV-ICD, caution is required if the LLP is an atrial pacing device, as atrial pacing may lead to inappropriate sensing, which needs to be evaluated at implant if being considered. VF induction is advised in these cases to confirm satisfactory function of both devices
	Dual-chamber leadless	Addition of leadless atrial device as part of Dual-Chamber AVEIR® system (Abbott, USA)^151^	Upgrading with an atrial leadless pacemaker may alleviate symptoms from leadless pacemaker syndrome. Success rates for deployment reported in a 36-patient series were 91.4% with complication rates reported to be 13.9%.^246–248^

Upgrade options include: (1) a leaded system (either a pacemaker, CRT, or defibrillator), (2) an S-ICD or EV-ICD, or (3) a dual-chamber leadless system.

*Table 11* outlines upgrade management and strategies for patients with an existing S-ICD (Boston Scientific, USA) or EV-ICD (Medtronic, USA). Upgrades may be appropriate if a patient develops a pacing indication, or if it is felt the individual patient would benefit significantly from ATP, or if the patient develops a resynchronization (i.e. LBBB and severe LV impairment) indication. Upgrade options include (1) a leadless pacemaker, (2) a leaded CIED (with removal of the S-ICD/EV-ICD if a TV-ICD is implanted), or (3) switch to EV-ICD from S-ICD or vice versa.

**Table 11:** Upgrade options and management strategies for patients with subcutaneous ICD (S-ICD) and EV-ICD

Existing CIED	Upgrade	Management strategies	Key points
S-ICD (Boston Scientific, USA)	S-ICD + LLP	For pacing needs, combining an S-ICD with an LLP may be preferred, particularly if elevated risk of CIED infection. This combination is more frequently encountered in complex congenital heart disease and/or if vascular access is limited/challenging^244,249–252^	During LLP deployment, S-ICD waveform screening should be performed for reasons detailed above. Consider repositioning of the LLP in case of S-ICD sensing concerns^244,245^ Confirm appropriate S-ICD sensing during VOO at high pacing output.^244,245^ Consider modular cardiac rhythm management (EMPOWER), when commercially available, as this will provide ATP functionality
	S-ICD + leaded CIED	For pacing indications, conventional transvenous leads are commonly used. However, if there are vascular access issues, an epicardial pacing system is a potential option. If ATP functionality is desired, a transvenous ICD lead can be implanted. In this scenario, the S-ICD should be removed If no ATP requirement but only Brady or CRT requirements, a pacemaker or CRT-P could be implanted and the S-ICD left *in situ*. If vascular issues are not present, it may be appropriate to implant an entire TV system and remove the S-ICD. Combining a TV CIED and an S-ICD may be used when vascular access is not sufficient for multiple lead implantation	Intraoperative S-ICD waveform screening during maximal voltage output and inhibition should be performed for reasons detailed above.^253^ Set the pacemaker in VOO mode at maximum output to watch for oversensing and also for VF under-sensing during defibrillation threshold testing in patients (i.e. test the ‘worst-case scenario’)^254^ Consider programming the pacemaker upper rate to <50% of the S-ICD tachycardia zone (avoids inappropriate therapy due to double detection) if oversensing appears to be a concern Obligate bipolar pacing output to avoid inappropriate double detection of pacing stimulus and evoked response Consider pacing approaches that deliver narrower QRS morphologies to reduce double counting risk^255^
	Replace with EV-ICD	This could be an option if ATP functionality is desired, no anti-bradycardia pacing indication and there is no venous access for a TV-CIED	Technology is in relative infancy. The pivotal study (*n* = 356 enrolled) has reported high defibrillation success rates and low complication rates.^149^ More data are needed on this technology
EV-ICD	EV-ICD + LLP	For pacing needs combining an EV-ICD with an LLP may be preferred, particularly if elevated risk of CIED infection or vascular is limited/challenging	Technology is in relative infancy. Atrial pacing may lead to inappropriate sensing and interaction will need to be tested at implant if being considered. Experience is limited to date^148,256^
	EV-ICD + leaded CIED	For pacing indications, conventional transvenous leads are commonly used. However, if there are vascular access issues, an epicardial pacing system is a potential option. If only Brady or CRT requirements, a pacemaker or CRT-P could be implanted, and the EV-ICD left *in situ*. If vascular issues are not present, it may be appropriate to implant an entire TV system and remove the EV-ICD. Combining a TV CIED and an EV-ICD may be used when vascular access is not sufficient for multiple lead implantation	Technology is in relative infancy. Atrial pacing may lead to inappropriate sensing and interaction will need to be tested at implant if being considered. Experience is limited to date^148,257^
	Replace with S-ICD	This could be an option if vascular access is limited/challenging and an alternative defibrillation strategy is desired	Maintains a non-transvenous and extravascular system

**Table of Advice:** Distinct case scenarios—CIED upgrade

CIED upgrade	Strength
**Advised to do**	
It is advised that when transvenous or leadless pacemakers are combined with subcutaneous or extravascular defibrillators, possible interactions between the systems are considered. Evaluation before and during implant should aim to reduce risk of inappropriate defibrillation delivery or inappropriate inhibition of pacing	>90% of writing group agree
When a CIED with an RV pacemaker lead is upgraded to a system with a transvenous defibrillator lead or to a conduction system pacing lead, considering the pros and cons of extraction over abandonment of the original RV lead is advised	>90% of writing group agree
It is advised when upgrading an existing pacemaker to a CSP system (no defibrillator) to avoid abandoning leads, and the already implanted RV lead can be incorporated into a CRT-P generator	>90% of writing group agree
**May be appropriate to do**	
It may be useful where a defibrillator lead is to be added, and there is a short lead dwell time of the original RV lead and/or younger age of the patient, that a lead extraction strategy is preferred over lead abandonment	>90% of writing group agree

## Device downgrade considerations

The decision to downgrade from a device capable of defibrillation has several considerations. Decision-making should begin with a careful evaluation of patient frailty, comorbidity burden, life expectancy, and adequately informed personal preferences. In patients with significant competing risks from non-arrhythmic causes of death or limited functional reserve, the benefits of continued defibrillator therapy may be outweighed by its potential burdens. As emphasized in the recent EHRA consensus document,^146^ shared decision-making is critical in these scenarios, ensuring that choices align with the patient's overall goals of care and values.

Beyond these core considerations, other factors may provide additional context for decision-making at the time of generator replacement. These include recovery of LVEF, the burden of ventricular arrhythmias, the presence or absence of an arrhythmogenic substrate (such as myocardial fibrosis or genetic abnormalities), and a patient's history of ICD therapies. While such elements can support clinical judgment in selected cases, they are largely informed by retrospective analyses and observational data, and no validated risk stratification tool currently exists to guide these decisions with confidence. As a result, the predictive value of these markers remains uncertain and should be regarded as adjunctive rather than definitive. The decision to continue or withdraw defibrillator therapy requires individualized, patient-centred assessment.

In a meta-analysis aiming to determine the effect of LVEF recovery following CRT implantation on the incidence of appropriate ICD therapy, Chatterjee *et al.*^258^ reported that LVEF recovery to ≥45% had a lower estimated incidence of ICD therapy compared to those who did not recover (2.3 vs. 8.2 per 100 patient years, *P* < 0.001). Therefore, although the risk of appropriate ICD therapy was not nil, it was below the estimated arrhythmic risk threshold of 3 per 100 patient years for ICD benefit, leading to a number needed to treat of 50 patients. If a blanking period for evaluating ventricular arrhythmias was applied between CRT implantation and diagnosis of LVEF recovery, the estimated arrhythmic risk was even lower at 1.7 per 100 patient years. Using an LVEF recovery cutoff of ≥35%, the estimated incidence of ICD therapy was 5.4 per 100 patient years (i.e. above the estimated threshold for benefit).

It is well established that patients with a secondary prevention indication are more likely to receive appropriate ICD therapy than those with a primary indication during the course of follow-up.^259^ However, it is unknown to what extent patients with secondary prevention are still at risk for sudden death after having recovered their LVEF with CRT.

Despite recovery of LVEF, there may be underlying substrates that maintain patients’ arrhythmic risk, such as genetic abnormalities (e.g. laminopathy^260^) or substrate detected by MRI, such as grey-zone myocardial fibrosis in ischaemic heart disease (an admixture of fibrosis and viable tissue),^261^ or mid-wall fibrosis in non-ischaemic cardiomyopathy.^95^

### Downgrade of cardiac resynchronization therapy defibrillator to cardiac resynchronization therapy pacemaker

The evidence for downgrading CRT-D to CRT-P is currently limited to two series totalling 73 patients with a primary prevention indication, no appropriate ICD therapy and LVEF improvement to >45%^262^ or >50%.^263^ There were no significant ventricular arrhythmic events over a mean follow-up of ∼4 years. Despite the arguments for downgrading from CRT-D to CRT-P, respondents to an EHRA survey reported that they performed these downgrades in <10% of patients.^264^ Furthermore, we still lack data from RCTs on this topic.

#### Practical considerations

**Figure 14:**
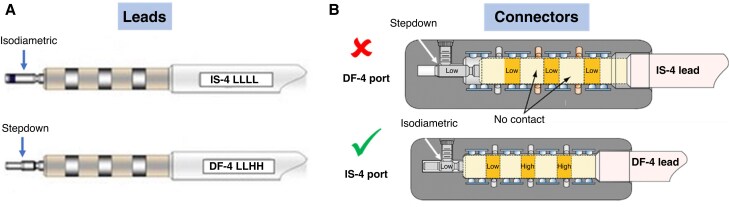
*A.* Lead pin design of IS-4 leads and DF-4 leads. Note the step-down in diameter of the DF-4 lead pin tip *B.* Connector design of DF-4 and IS-4 ports. Note the step-down in DF-4 connector diameter, which prevents the isodiametric IS-4 lead from being fully inserted. Conversely, the DF-4 lead fits into the isodiametric IS-4 port.

Unlike the DF-1 ICD connection standard, where downgrade from CRT-D to CRT-P can be easily achieved by simply abandoning the DF-1 pin(s) and connecting the IS-1 pin to the pacemaker generator, the DF-4 standard is more challenging due to the low- and high-voltage components all being integrated together on the same pin (with additional limitations, as covered elsewhere.^1^ The issue could be solved by a DF-4/IS-1 adaptor, which is unfortunately currently unavailable. The following options are possible though:

1. *The simplest approach involves a like-for-like generator replacement and simply deactivating the ICD capabilities of the new device at the time of generator replacement*. Unfortunately, this is not a particularly cost-efficient upfront solution for these patients but has minimal additional risk.
2. *Implanting a new ventricular pacing lead and abandoning the DF-4 lead*.
3. *Extracting the DF-4 lead and replacing with a ventricular pacing lead*. This strategy carries the inherent risks of lead extraction and the additional risk of damaging or dislodging the CS lead in case of lead-lead adhesions. The cost of extraction and its possible complications, especially in frail patients, likely outweighs the cost of replacement with a defibrillator device.
4. *Conversion of a DF-4 ICD lead to a unipolar IS-1 lead has recently been described in four patients in abstract-form only. This approach uses an Oscor M/IS-10 5 mm to IS1 adaptor and Abbott 4033A lead cap. Experience with this is limited and the long-term durability of this modified lead is unknown* ^265^
5. *Simple lead switch on the CRT-P header in patients with a DF-4 lead and a bipolar CS lead*.^266^ It is possible to connect the DF-4 pin to the IS-4 port (of the CS pacing lead), but not vice versa—see *Figure 14*. This is a safety feature to prevent shocks being delivered via an inadvertently switched pacing lead in a DF-4/IS-4 CRT-D device. However, as the great majority of CS leads currently are of the IS-4 standard, this is rarely an option for CRT-D cases.
6. *Utilization of an Abbott CRT-P, which allows LV sensing*. It is possible to cap the DF-4 component, which is not required, and simply connect an IS-4 quadripolar LV lead to the LV port, which will enable delivery of LV-only pacing. Retaining MRI-conditionality in this scenario is a labelling issue.

### Downgrade of implantable cardioverter defibrillator to pacemaker

In this scenario, patients have a pacing indication (otherwise, a CIED would no longer be indicated) with either infrequent requirement for pacing or relatively preserved ejection fraction (otherwise, CRT would be preferable). Using a cutoff for LVEF of >40–45% is prudent, bearing in mind the variability of LVEF measurement (even if most indications for ICDs are LVEF <35%).^258^ Programmed ventricular stimulation may be performed in selected cases (via the transvenous ICD), aiming to improve risk stratification of sudden death and continued ICD indication, although this strategy has never been tested.

#### Practical considerations

As described above, the DF-4 lead may be connected to the IS-4 port of a CRT-P, with plugging of the RV port. However, currently, only Abbott (Sylmar, USA) CRT-Ps are enabled with bipolar sensing from the LV channel with resetting of standard timing cycles. Boston Scientific (Marlborough, USA) and Biotronik (Berlin, Germany) CRT-Ps are also capable of sensing from the LV channel, but this is only intended to prevent LV pacing in the vulnerable period after a left-sided ventricular extrasystole and does not impact right-sided timing cycles, which makes them unsuitable for downgrades in instances without an RV pacing lead. A case series of three patients with this strategy has been published.^267^ MRI-conditionality is lost when the DF-4 lead is plugged into the LV port, but this configuration is unlikely to put patients at risk for MRI scans (although no data are available).

**Figure 15:**
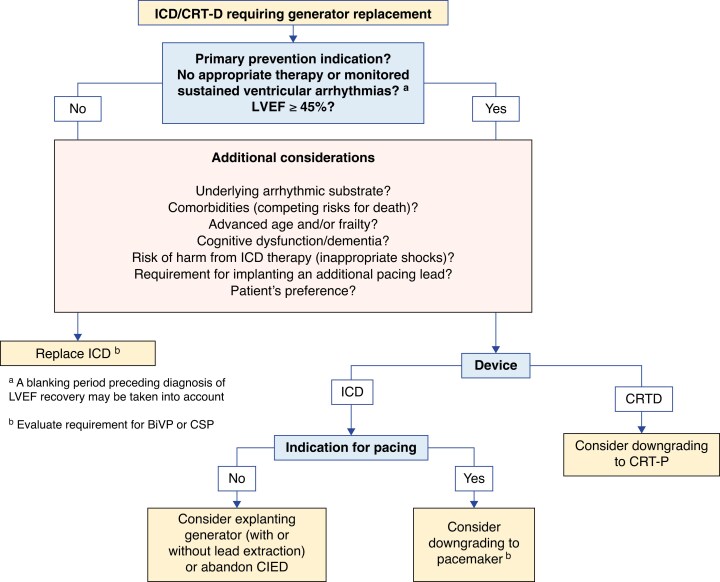
Decision tree for consideration of downgrade of CRT-D or ICD at ICD generator change. ICD, implantable cardioverter defibrillator; CRT-D, cardiac resynchronization therapy defibrillator; CRT-P, cardiac resynchronization therapy pacemaker; LVEF, left ventricular ejection fraction.

A decision tree for evaluating options at ICD generator change is outlined in *Figure 15* and highlights when downgrade may be a suitable management approach.

### Downgrade from dual-chamber to single-chamber device

In patients with dual-chamber devices who develop permanent atrial fibrillation, the atrial lead becomes redundant. At generator change, the following options are available:

1. *Replace with a dual-chamber generator.* This avoids abandoning the atrial leads, thus retaining MRI-conditionality, which may be the preferred strategy in most patients, particularly those requiring MRI.
2. *Replace with a single-chamber device and abandon the atrial lead.* MRI-conditionality is thereby lost. In the same manner that the ESC pacing guidelines allow MRIs to be performed in selected pacemaker patients with abandoned leads, an EHRA consensus document similarly allows MRI in ICDs with abandoned leads.^268^ Furthermore, it is unknown if abandoned atrial leads are associated with the same potential risks as abandoned ventricular leads (as induced atrial arrhythmia is not an issue in patients with permanent atrial fibrillation).
3. *Replace with a single-chamber device and extract the atrial lead.* This has the advantage of avoiding any abandoned material but exposes the patient to the risks of lead extraction.

### When a cardiac implantable electronic device is no longer indicated

Different options are available for patients in whom a CIED is no longer indicated:

1. *Abandon the CIED.* The end-of-life behaviour of CIEDs is device-specific and may vary from erratic behaviour (with potential pro-arrhythmia) to safe inactivation. Inactivating and abandoning the CIED may be the preferred option in frail and elderly patients.^13^
2. *Explant the generator and leave the lead(s) in situ.* This avoids consequences of end-of-life erratic CIED behaviour but precludes MRI-conditionality and additionally carries a degree of procedural risk (namely infection) from re-intervention on the generator pocket. However, generator explant has an additional advantage in particularly frail, sarcopenic, or cachectic patients, where a redundant generator may excessively impact upon skin integrity
3. *CIED extraction.* This may be the preferred option in young patients as it avoids all future device-related complications but comes with the highest initial risk.

Individual risk–benefit analysis should be performed in each case with shared decision-making with the patient and caregivers. Some considerations in such risk–benefit analysis are currently unknown including for example, the value of programmed ventricular stimulation via the ICD before generator replacement in patients with LVEF > 35%, to stratify risk of sudden death and evaluate requirement for continued ICD therapy or the risks with MRI in the setting of abandoned atrial leads (without abandoned ventricular leads), abandoned DF-1 components of high-voltage leads, DF-4 leads plugged in IS-4 ports and abandoned leads which are capped vs. those which are sectioned.

**Table of Advice:** Distinct case scenarios—CIED downgrade

CIED downgrade	Strength
**Advised to do**	
At the time of elective generator replacement, it is advised to undertake an evaluation of the ongoing need for defibrillator therapy. Consideration to potential device downgrade—such as from CRT-D to CRT-P, from ICD to a pacemaker, or deactivation or explant of the ICD may be appropriate. Factors to prioritize to guide the decision include patient frailty, comorbidities, life expectancy, and informed patient preference. All considerations should be integrated into a shared decision-making process tailored to the patient's overall clinical context and goals of care^262,263^	>90% of writing group agree
When pacing is no longer indicated, it is advised that the management strategy is based on an individual risk–benefit analysis in a shared decision-making process together with the patient and caregivers	>90% of writing group agree
**May be appropriate to do**	
At generator change in a patient with a dual-chamber pacemaker or ICD who has developed permanent atrial fibrillation, replacement with a dual-chamber device to retain MRI-conditionality may be appropriate (rather than utilizing a single-chamber device and abandoning the atrial lead)	>90% of writing group agree
**Areas of Uncertainty**	
In patients with primary prevention ICDs who have not previously had device therapies, the optimal approach to determine the arrhythmic risk at the time of generator replacement is uncertain. This risk stratification might inform who can be safely downgraded.	>70% of writing group agree
The safety of downgrading CRT-D to CRT-P in patients with secondary prevention indications with recovered LVEF (>40–45%), without appropriate ICD therapy, remains uncertain	>90% of writing group agree
The prognostic significance of ventricular arrhythmias occurring only early after CRT-D implantation, before recovery of LVEF has resulted (which may constitute a blanking period) remains uncertain	>90% of writing group agree

## Distinct patient scenarios

*Table 12* provides case vignettes and example management options and strategies for CIED upgrade and downgrade patient scenarios.

**Table 12:** Case vignettes and example management options and strategies for CIED upgrade and downgrade patient scenarios

Patient scenario	Management strategies and options	Key points
A 63-year-old man with ischaemic cardiomyopathy and a dual-chamber ICD implanted 10 years ago for primary prevention (LVEF 30%). He develops VT storm and is treated with Amiodarone, which increases his ventricular pacing percentage to >20%. He presents with worsening heart failure and a deterioration of his LVEF to 20%. He is referred for an upgrade to a CRT-D. His venogram reveals his axillary vein is occluded	Given the high prevalence of venous stenosis in patients with existing CIED leads, a peripheral venogram was performed, which confirmed axillary vein occlusion. Management strategies were discussed, including medial subclavian puncture, attempts at re-establishing vascular patency via ipsilateral venoplasty or lead extraction, vs. lead abandonment and re-implant of a new CRT-D on the contralateral side, or implantation of upgrade lead on contralateral side and tunnelling to existing pocket The patient opted to attempt re-establishing vascular patency first by venoplasty, and if that failed, lead extraction and re-implantation would have been used to facilitate completion of the upgrade procedure at the same sitting to avoid multiple procedures increasing risk of infection A successful upgrade procedure was performed after venoplasty with addition of an IS4 LV lead	A consideration in this case is his relatively young age and possibility of risks related to lead abandonment if an entire *de novo* system were to be implanted on the contralateral side Attempts at re-establishing vascular patency with venoplasty are relatively low risk and frequently successful even when veins appear occluded. Regaining vascular access via lead extraction may have a higher risk than venoplasty, but it is an effective alternative if venoplasty fails. Lead abandonment and potential future risk of lead extraction, which might increase with higher lead burden and dwell time (for example in the scenario of if abandoned leads become involved in a later infected device)
An 82-year-old woman with permanent atrial fibrillation, renal impairment, and diabetes. VDD pacemaker implanted 9 years ago as part of an ablate and pace strategy. Inhibition of right ventricular pacing was noted due to the oversensing of myopotentials with ipsilateral arm movements Her venogram revealed chronic ipsilateral left subclavian and left brachiocephalic vein occlusion. No evidence of LV impairment or PICM was apparent even with her high burden of RV pacing	Management options discussed included attempting venoplasty to gain access for a new lead, extraction and replacement of ventricular lead, abandonment and addition of a new contralateral ventricular lead and device, or abandonment and implantation of a leadless pacemaker Ipsilateral venous occlusion would require contralateral lead placement, ipsilateral vein venoplasty, or lead extraction and re-implant of a new lead through the occluded vein. Considerations included the patient's preference for a simpler procedure with lowest expected complications. The decision was to abandon the malfunctioning lead and to implant a VVIR leadless pacemaker. The decision also included to remove the old pacemaker generator to avoid future risks from interactions with the new leadless pacemaker, for example, automatic programming in VOO at ERI or EOL	Key considerations were lack of evidence of PICM, preserved LV function, advanced age and patient preferences A leadless pacemaker reduces the likelihood of future CIED infections Only one abandoned pacing lead. Likelihood of future CIED interventions (the patient may not require further pacemaker replacements or interventions due to her life expectancy, thus decreasing further the possibility of CIED infection) It is important to note that the whole transvenous system could have been abandoned, potentially reducing procedural complication, but this would be more relevant if the original device could be programmed ‘OOO’ thus mitigating risk of device interaction
An 81-year-old woman with non-ischaemic cardiomyopathy had an ICD implanted 13 years ago (primary prevention). Dual coil and passive fixation ICD lead. No pacing indication During follow-up and on guideline-recommended heart failure medical treatment, the LVEF increased to 50%. No ventricular arrhythmias were observed during all follow-up visits. Frequent episodes of myopotential oversensing were observed during remote monitoring	Management options discussed included abandoning lead and generator, removing generator and abandoning the ICD lead, or extracting the ICD lead and removing the generator Reappraise ICD indication (primary prevention, no ICD therapies, LVEF improvement) Considerations included the patient’s preference not to have a prolonged hospitalization/recovery period and not wanting a redundant generator in the pocket The patient did not want to undergo extraction. At her request, the decision was made to remove the generator and abandon the lead. No new ICD was implanted	Currently, no indication for ICD implantation. Considerations are: Age of the patient The type of ICD lead (dual-coil and passive fixation), as well as long dwell time, increases the risk of lead extraction Abandoned leads are only a relative contraindication for MRI The patient remains at ongoing risk for lead infection, which, if occurred, would require a higher-risk extraction in the future Opening the pocket to remove the generator may have exposed the patient to a risk of infection However, leaving the ICD *in situ* could leave the patient at risk (likely low) of erratic EOL behaviour Consideration could have been given to abandoning the whole system, reducing procedural complication, and preserving MRI labelling
A 79-year-old woman with dual-chamber PPM for intermittent complete heart block. Recent generator replacement (leads 12 years old). Recent mitral valve replacement and tricuspid valve annuloplasty for severe MR and TR. Post-operative LBBB development. TR remains moderate to severe despite intervention and in part this is due to splinting of valve by existing RV pacing lead. LVEF has reduced to 23% post-surgery Significant collateral veins were seen on the chest wall and a venogram confirmed significant subclavian stenoses. Patient is 49 kg	Decision regarding upgrade of device to CRT-P vs. D and decision regarding upgrade to biventricular pacing vs. CSP Despite a recent generator replacement, it was appropriate to proceed quickly to device upgrade and not defer. Given her age, comorbidity, non-ischaemic aetiology, absence of any ventricular arrhythmia history and her elicited values with shared decision-making, upgrade to CRT-P with an LV lead only using venoplasty to gain adequate venous access was chosen	To avoid additional leads traversing the tricuspid valve the decision was made to not implant an ICD lead nor for a CSP lead. In view of her low BMI, sarcopenia and frailty an ICD was not favoured to avoid a large generator. This was the favoured approach particularly as ventricular arrhythmia risk was felt to be low. Evidence of benefit of LV leads alone in patients with non-ischaemic cardiomyopathy and broad LBBB is significant At the time of cardiac surgery, epicardial LV lead implantation could have been a potential alternative

## Conclusion

In this document, the key considerations (patient, procedural, and ethical) that should be evaluated in patients being assessed for device upgrade and downgrade procedures are summarized. We support the systematic ongoing evaluation of device type and suitability at all follow-up interactions. We highlight patient-specific risk factors that need to be accounted for when determining device upgrade or downgrade suitability, including, importantly, patient frailty. We provide an overview of available tools and techniques that can be used to safely and efficiently perform procedures, with a particular focus on how to achieve vascular access with discussion around the relative risks and benefits of lead abandonment and extraction approaches. We finally provide a multi-disciplinary framework for device downgrades that can be utilized in clinical practice. Knowledge gaps remain for definitive assessment as to whether any of the procedural techniques lead to superior patient outcomes over another technique. Furthermore, randomized evidence is required to help better refine which patient groups benefit most from the armamentarium of device upgrade options.

## Data Availability

No new data were generated or analysed in support of this research.
